# Prediction of clinical outcomes of ST-elevated myocardial infarction patients using atmospheric solids analysis probe mass spectrometry and machine learning

**DOI:** 10.1039/d5an00565e

**Published:** 2025-09-24

**Authors:** Annabel S. J. Eardley-Brunt, Thomas Mills, Rafail Kotronias, Giovanni Luigi de Maria, Keith Channon, The Oxford Acute Myocardial Infarction (OxAMI) study, Claire Vallance

**Affiliations:** a Department of Chemistry, University of Oxford, Chemistry Research Laboratory 12 Mansfield Rd Oxford OX1 3TA UK; b NIHR Oxford Biomedical Research Centre, John Radcliffe Hospital, Oxford University Hospitals Oxford UK; c Division of Cardiovascular Medicine, Radcliffe Department of Medicine, University of Oxford Oxford UK claire.vallance@chem.ox.ac.uk

## Abstract

*Introduction*: Analysis of small molecule metabolites found in blood plasma of patients undergoing treatment for STEMI has the potential to be used as a clinical diagnostic and prognostic tool, capable of predicting disease progression, risk of negative outcomes, and response to treatment. *Methods*: Rapid mass spectrometry has been used to measure the metabolite profiles of coronary aspirate blood plasma from 288 STEMI patients enrolled in the Oxford Acute Myocardial Infarction (OxAMI) study. Supervised machine learning applied to the mass spectra was used to stratify patients based on clinically relevant variables related to health and treatment response. *Results*: In this small proof-of-concept study, patient mortality and microvascular obstruction (MVO) were predicted with over 80% accuracy; heart failure diagnosis, ischemic time, peak troponin, and thrombus score were predicted with over 76% accuracy, and creatinine and index of microcirculatory resistance (IMR) were predicted with over 70% accuracy. Using feature-reduction methods, we were able to identify key mass-to-charge (*m*/*z*) peaks in the mass spectra that correlated with the assignment to particular patent groups. These may potentially be used in the future as mass spectrometric biomarkers in the development of a diagnostic and prognostic test for STEMI risk.

## Abstract 2

Rapid mass spectrometry is used to measure metabolite profiles of coronary aspirate plasma from heart attack patients. Supervised machine learning is used to categorise the data based on clinically relevant variables quantifying cardiac health.

## Graphical abstract

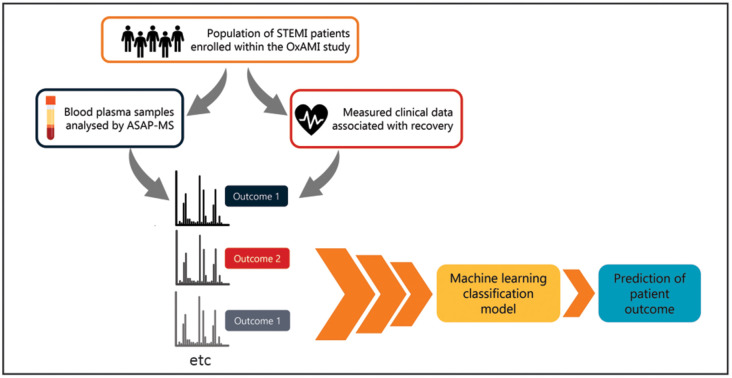

## 1 Introduction

ST-segment-elevation myocardial infarction (STEMI) is a severe yet common clinical condition that continues to be associated with a high risk of patient mortality.^1^ From 2021 to 2022, over 85 000 confirmed acute myocardial infarction (AMI) cases were reported by the National Cardiac Audit Programme in the UK, 36% of which were the higher-risk STEMI, and the remaining 64% being the lower-risk non-ST-segment-elevation myocardial infarction (NSTEMI).^2^ Current treatments and advances in hospital care have lead to a gradual decrease in mortality of AMI patients.^3^ However there remain a high number of cases in which mortality occurs in the two years post-presentation due to complications including subsequent heart failure. In addition, patient readmission as a result of cardiovascular episodes and related diseases remains a significant burden on healthcare systems.^4–6^ Identification of ‘high risk’ patients earlier on in their treatment journey would allow a more targeted response to their initial STEMI, employing more aggressive treatments that are withheld from the general patient population due to a high risk of complications, cost, or unpleasant side effects. The aim of the present study is to determine whether biological markers present in coronary aspirate blood plasma of STEMI patients can prove predictive for their progression and recovery trajectory. Classification of patients into risk categories for disease progression will allow for more personalised, targeted treatments that may prolong or enhance the lives of patients with cardiovascular disease.

Due to recent improvements in the availability, collection and publication of large clinical datasets, an influx of studies have been published over the last three years that utilise machine learning and multivariate statistics to predict outcomes for STEMI patients.^7–12^ Some of the models developed within studies include data for thousands of patients, in the form of both demographic information (age, sex, family history) and clinical parameters (thrombus score, IMR, troponin), and can predict negative patient outcomes such as mortality with high accuracy. The OxAMI data set has been used previously to develop models of this type, with varied results.^13–20^ These various studies have led to the development of a range of functions and composite scores; examples include the age-thrombus burden index of microcirculatory resistance (ATI) score and resistive reserve ratio (RRR), both of which show prognostic value for STEMI patients.^18–20^ Based on these results, it is clear that indicative predictions of probable patient outcomes and response to treatment are possible based on early patient information. Within the present study, we take this relationship closer to the pathology of the disease, by linking the prediction of patient outcomes directly to a measurement of metabolites in the blood.

Achieving an accurate representation of the biochemical composition of complex biological matrices such as blood plasma is challenging. Most analytical instruments report on a selected fraction of the matrix with known physiochemical attributes, *e.g.* ‘lipophilic molecules’, or require the use of various mechanical and chemical separation steps involving the addition of solvents that may alter the sample. Recent developments in ion-source technology have led to the introduction of a range of analytical instruments that can measure complex samples in their native state under atmospheric conditions, and in some cases *in vivo*. Ambient ionisation mass spectrometry has enabled the rapid analysis of biological samples in a clinical setting, and has been employed in a diverse range of applications, from tumour differentiation and margin delineation to quantitative analysis of pharmaceuticals in blood micro-samples.^21–24^ Atmospheric solids analysis probe mass spectrometry (ASAP-MS) is a form of ambient ionisation instrument capable of performing untargeted profiling of the small-molecule composition of solid and liquid biological samples in their native state.^25–27^ Measurements are completed in a matter of seconds, making these compact instruments a potential candidate for use in a clinical setting. The method provides an untargeted ‘molecular fingerprint’ of the sample, allowing for a pattern recognition approach to biomarker discovery to be conducted. This approach complements conventional specific biomarker discovery methods in a number of ways. Firstly, there is no requirement for comprehensive knowledge of the biochemical pathways and specific molecules underpinning the disease, saving time as well as costs associated with multi-omics analysis studies. Secondly, many biomolecules may be measured simultaneously, providing an opportunity to identify subtle relationships between metabolites that may otherwise be missed, and potentially opening studies up to include biomarkers beyond the current known molecular space. In the case of STEMI metabolomics, an untargeted approach may shed some light on the relationship between metabolic pathways for lipophilic and non-lipophilic molecules, with (amongst others) both glucose and lipid metabolism known to play a crucial role in myocardial ischemia-reperfusion injury.^28^

The pattern recognition process required to link mass spectrometric fingerprints with clinical outcomes may be performed in a number of ways, with some of the most promising being machine-learning approaches.^29^ A variety of machine learning algorithms have been developed to conduct pattern recognition or classification analysis on multivariate data sets, including collections of mass spectra.^29–32^ These can be employed in the present context to classify patients into risk groups based on correlations between the mass spectra and a number of clinical parameters known to be linked with further complications or patient mortality. Machine-learning methods can also be combined with feature reduction techniques in order to determine which features of the mass spectra, *i.e.* which molecular signals, are most important in the classification process. Such approaches identify which metabolite mass peaks show significant differences between the patient groups, and might therefore be worthy of further investigation as potential biomarkers.

In the present study, we have investigated the use of machine learning to analyse mass spectra of coronary aspirate blood plasma from STEMI patients enrolled on the OxAMI clinical study,^33,34^ with the goal of stratifying patients according to their future cardiac risk. The coronary aspirate samples were collected during the primary percutaneous coronary intervention (pPCI) undergone by patients on admission to hospital, and were chosen for this initial proof-of-concept study as we hypothesised they were likely to contain the highest concentrations of metabolites related to the coronary event. The coronary aspirate is a waste product of the cardiac catheterisation procedure, and is usually disposed of. While mass spectrometric analysis of clinical samples has become a key tool for the generation of models predicting clinical outcomes in various disease states, including in cardiovascular diseases,^35–39^ we believe this is the first application of ASAP-MS in the prediction of outcomes for any disease. ASAP-MS provides results much more rapidly than alternative high-resolution-omics techniques, as well as having low resource and training requirements. This makes it potentially suitable for use within a clinical setting, though we note that the relatively low *m*/*z* resolution means that obtaining detailed molecular assignments for the *m*/*z* signals would require measurements with a higher resolution technique over the mass ranges of interest. For these reasons, we will focus on the ability of ASAP-MS coupled with machine learning methods to classify patients into appropriate risk groups, rather than on biomarker discovery.

## 2 Methods

**Fig. 1:**
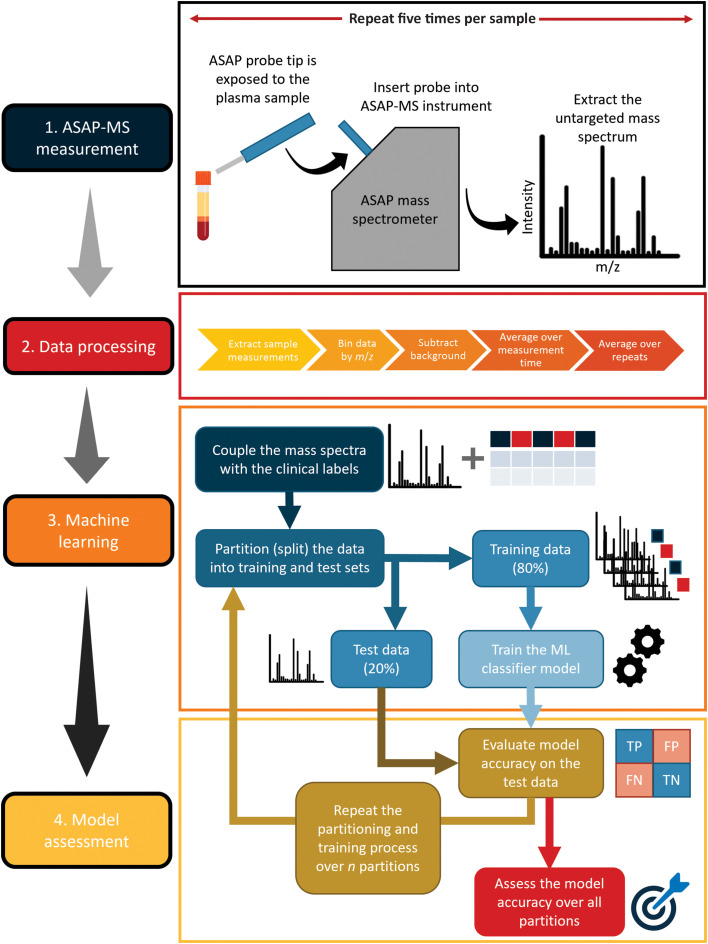
Summary of the workflow employed in the present study. See text for further details.

The overall workflow for the study is summarised in Fig. 1. We describe each of the steps in more detail in the following.

### 2.1 Study patients and criteria

The 283 coronary aspirate plasma samples used in this study were acquired from STEMI patients enrolled in the Oxford Acute Myocardial Infarction (OxAMI) clinical study, who underwent primary percutaneous coronary intervention (pPCI) at the John Radcliffe Hospital, Oxford, between 2010 and 2021. We were granted access to a set of samples in order to investigate the utility of ASAP-MS fingerprinting for prognostic purposes, as a small early-stage side project within the primary clinical investigations of the study. It should be emphasised that the pilot metabolomic fingerprinting analysis performed within the present study is a secondary use of the samples, and not the primary clinical study objective for the overall OxAMI project. The selection criteria for patients enrolled in the OxAMI study have been described in detail previously.^40–42^ Briefly, STEMI was identified by chest pain continuing for >30 min and ST elevation >2 mm in >2 contiguous leads. Exclusion criteria were symptom duration >12 h, the presence of severe haemodynamic instability, and contraindication to the use of adenosine. The study was conducted in accordance with the Declaration of Helsinki. All participants gave written informed consent (REC number 10/H0408/24).

The coronary aspirate blood samples were obtained during pPCI to restore blood flow to the affected coronary artery. The plasma component of each sample was isolated by mechanical separation using a centrifuge, snap frozen in dry ice, and stored in a freezer at −80 °C until measurements were made.

Data available for patients enrolled in the OxAMI study include standard medical demographic information (*e.g.* age, sex), health and lifestyle indicators (*e.g.* weight, smoking, diabetes, blood pressure), and measured clinical parameters relevant to the presentation and treatment of the STEMI. Table 1 shows the distribution of patients across the various demographic and health indicators. We note the highly uneven proportions of patients in each category for some parameters, including gender and body mass index (BMI), a direct consequence of the various well known risk factors for myocardial infarction (MI).

**Table 1:** Demographic information for patients enrolled in the OxAMI study. Columns 2 and 3 give the number of patients in each demographic group, while column 4 gives the percentage of patients enrolled in the OxAMI study for which the relevant data are unavailable. Full details may be found in earlier publications from the OxAMI study^40–42^

Clinical parameter	Group 1	Group 2	% missing
Patient sex	Female, 48	Male, 217	6.36
Previous cardiological history	No, 214	Yes, 106	6.36
Current smoker	No, 160	Yes, 106	6.01
Ex-smoker	No, 117	Yes, 106	29.68
Smoker or ex-smoker	No, 78	Yes, 184	7.42
Hypertension	No, 154	Yes, 112	6.01
Diabetes	No, 226	Yes, 40	6.01
Hypercholesterolemia	No, 165	Yes, 100	6.36
Family history of MI	No, 157	Yes, 109	6.01
Previous MI	No, 249	Yes, 16	6.36
Previous pPCI	No, 250	Yes, 15	6.01
Peripheral vascular disease	No, 134	Yes, 5	50.88
Chronic obstructive pulmonary disease or Asthma	No, 125	Yes, 16	50.88
Age over 60	No, 108	Yes, 155	7.07
BMI over 25	No, 64	Yes, 174	15.90

### 2.2 Measurement protocol

Frozen blood plasma samples with volumes of between 0.2 and 2 mL, stored at −80 °C in 2 mL micro tubes, were obtained from the Oxford Heart Centre, John Radcliffe Hospital. Mass spectra of blood plasma were recorded on an Advion expression® compact mass spectrometer ASAP-MS instrument, equipped with Advion Mass Express data acquisition software and Advion Data Express data manipulation software (both version 6.9.38.1). The detailed experimental protocol used in our laboratory for plasma ASAP-MS measurements has been published previously,^43^ along with steps that can be taken to maximise data quality for clinical data sets recorded *via* ASAP-MS.^44^ When following the protocol summarised below, we typically achieve a coefficient of variance below 40% for plasma measurements.^43^

**Fig. 2:**
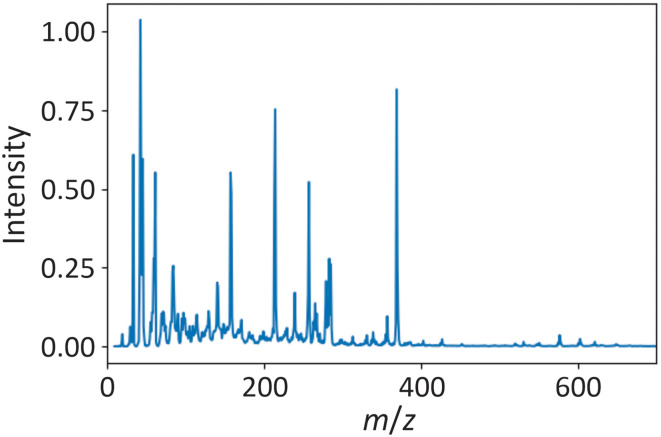
Example of an ASAP mass spectrum recorded for one of the OxAMI plasma samples.

Prior to the measurements, the glass capillaries (Advion ASAP S01 short) that form the tip of the ASAP probe used to load the samples into the instrument (see section 2.2) were sterilised by heating in an oven for 30 minutes at 250 °C, and then stored in a desiccator. At the start of each day, instrument calibration was conducted using Advion APCI calibration/tune standard mix. All subsequent measurements were made with the ion source set to ‘high temperature, low fragmentation’ positive ion mode. Initially, following calibration, the instrument was operated with an unloaded probe for 30 min to allow the background signal to stabilise. For each sample, a clean capillary was loaded into the ASAP probe and used to record a background measurement for 30 s. The probe was then removed from the mass spectrometer, and the tip cooled with a small amount of methanol and dried with lens tissue before loading the sample. Prior to measurement, each sample was thawed at room temperature and then mixed by vortex mixer for 15 seconds to obtain a homogeneous sample. Only a tiny amount of sample is needed for an ASAP-MS measurement; a suitably small amount of sample was transferred to the probe tip by sampling from the internal surface of the sample tube just above the level of the plasma surface. The probe was then inserted into the ASAP-MS instrument and data acquired for 25 s, before the probe was removed, cleaned with methanol, and wiped dry with lens tissue. The sampling, measurement, and cleaning steps were repeated four more times using the same capillary for a total of five measurements per sample. The capillary was then discarded and a fresh capillary inserted for the next sample. In between measurements, the total ion count was monitored to ensure return to the baseline level before a new sample was introduced. An example mass spectrum recorded for one of the OxAMI plasma samples is shown in Fig. 2.

### 2.3 Data processing

Raw data files were exported for analysis *via* the Advion Data Express software and processed using a Python 3.7 script. As explained above, five sample measurements are made on each acquisition. However, the mass spectrometer completes a scan every 900 ms during the entire acquisition period, and the raw data files contain all of these scans stored in sequence. Some of these scans correspond to ‘background’ recorded when the probe was not present in the ion source, and some correspond to signal recorded during one of the five 30 s intervals when the probe loaded with sample was inserted into the ion source. The Python script: (i) identified these regions based on the rapid rise in the total ion count on each insertion of the probe; (ii) calculated the average and standard deviation of the mass spectra recorded over each 30 s sample measurement; and (iii) binned the mass spectra to unit mass in order to reduce the file size and remove the need for precise spectral alignment to be performed. An equivalent background spectrum was generated using the 30 s background measurement (see section 2.2), and subtracted from each of the five ‘signal’ measurements. The five background-corrected spectra were then normalised to unit area under the spectrum, and averaged to generate a single mass spectrum for each sample. We note that we have recently carried out a detailed study (to be published separately^45^) in which we used the data set recorded in the present study to compare a wide variety of different normalisation methods, including quantile, vector, and Pareto scaling approaches. We concluded that the choice of normalisation protocol had only a very small effect on the outcomes of the data analysis described in the following section, and that simple area-under-the-curve normalisation is entirely satisfactory in this context.

### 2.4 Data analysis

As noted above, the data analysis was designed to investigate correlations between the ‘molecular fingerprints’ contained within the plasma mass spectra, and a number of clinical parameters of interest,^40–42,46,47^ as detailed in Table 2. Five standard supervised machine learning classification models were chosen in order to investigate correlations between the plasma mass spectra and relevant clinical parameters, based on an evaluation of each algorithm's robustness, suitability across a range of data types, and ease of use. The K-nearest neighbours^48^ (KNN), support vector machines^49^ (SVM), and linear discriminant analysis^50^ (LDA) algorithms treat the mass spectral data as vectors in *N*-dimensional space (with *N* the number of data points, *i.e.* mass bins, in each mass spectrum), and classify mass spectra into categories either by considering the distances between vectors, projecting them onto a new set of axes, or finding multidimensional surfaces that separate them into classes. The naïve Bayes classifier (NBC) algorithm^50^ is a probabilistic model based on Bayesian statistics, while the random forest classifier^51^ (RFC) model uses decision trees to assign each mass spectrum to the appropriate class. These classical machine-learning methods can be considered to be closer to multivariate statistics than to more sophisticated methods such as deep neural networks, and are suitable for use with the relatively low numbers of samples available in the present study.

**Table 2:** Categories used for the binary classification of mass spectra according to the clinical variables of interest in the OxAMI study. Columns 2 and 3 give the criteria by which patients are assigned to each category. Column 4 gives the number *n* of patient samples available for each category (the total number of patients for each clinical variable is 2*n*). Where relevant, column 5 gives the names of the reduced data sets containing sample data for patients with extreme values of the corresponding clinical variable

Clinical parameter	‘Low’ (class label 0)	‘High’ (class label 1)	*n* patients per class for ‘all patients’ models	Data set names for ‘extreme patients’ models
Patient died	No	Yes	23	N/A
Heart failure diagnosis (HFD)	No HFD	HFD	25	N/A
Microvascular obstruction(MVO)	MVO = 0	MVO > 0	64	MVO-20, MVO-30
Index of microcirculatory resistance (IMR)	IMR < 40	IMR > 40	63	IMR-20, IMR-30
Thrombus score (TScore)	TScore 1–3	TScore 4–5	45	TScore-20, TScore-30
Ischemic time (ITime)	<6 hours	>6 hours	46	ITime-20, ITime-30
Peak troponin	<50	>50	110	Troponin-20, troponin-30
Creatinine on admission	<75	>75	121	Creatinine-20, creatinine-30

Further details of the machine learning models can be found in the SI. All data analysis was conducted using custom MATLAB R2022a and Python 3.7 software. Machine learning analysis was run on CPU nodes within the University of Oxford Advanced Research Computing cluster.

The numbers of patients involved in the OxAMI study, and therefore the number of samples in the mass spectral data set, were too low to perform the regression analysis required in order to predict numerical values for each clinical variable. Instead, patients were assigned as having ‘high’ or ‘low’ values for each clinical parameter of interest, based either on thresholds defined in the literature, or using the median measured value (see SI for details). The thresholds employed for each clinical variable are shown in Table 2. Having defined these thresholds, we were then able to investigate the extent to which machine learning classification algorithms could assign patients correctly to each group based on the mass spectra for their respective plasma samples.

To ensure that the machine learning algorithms use features of the mass spectra, *i.e.* mass-to-charge (*m*/*z*) peaks, to perform the classification, rather than ‘learning’ based on the balance of probabilities, it is important to ensure that the data set used to train the algorithm contains a similar number of mass spectra for each group in the classification. As a consequence, the mass spectra used to train the algorithms were generally a subset of the total available number of mass spectra. For example, our data set contained samples from 25 patients diagnosed with heart failure and 200 with no heart-failure diagnosis, as well as 58 patients for whom this diagnosis was not recorded. When constructing a classifier to distinguish between ‘heart failure’ and ‘no heart failure’ groups, we should include mass spectra for samples from 25 patients in each group, rather than using all 200 mass spectra available for the ‘no heart failure’ patients. This way the algorithms are forced to use features of the mass spectra to predict heart-failure status, rather than ‘learning’ that simply guessing ‘no heart failure’ will give a correct result over 85% of the time.

The number of patients per class is shown in Table 2 for each of the clinical parameters investigated. The available mass spectra were partitioned into training and test data in an 80 : 20 ratio, with the training data used to train the machine learning models, and the test data used to evaluate the performance of the models. The specific choice of training and test data from the overall data set can sometimes result in rather misleading overperformance or underperformance of a model. To avoid this, a cross-validation procedure was employed, in which the training and evaluation process was repeated multiple times with different selections of mass spectra allocated to the training and test sets on each run (see SI for further details).

For a clinical parameter that can take continuous values, setting a somewhat arbitrary threshold between ‘high’ and ‘low’ is unlikely to result in clearly separated groups: while we expect samples from patients at the extreme ends of the distribution to exhibit significant differences, the same is not true in the region of the threshold. To explore this, in addition to the complete mass spectral data set (referred to as ‘all patient data’ in the remainder of the paper), we also prepared ‘extreme patient’ data sets. These comprised subsets of mass spectra for patients with extreme high and low values for each variable. In the context of this early pilot study, this approach offers the best chance of identifying mass spectral differences correlating with the clinical variable of interest. If successful, further investigation with larger data sets, broader distributions, and potentially more sophisticated machine-learning approaches may be warranted. However, if classification is unsuccessful for ‘extreme’ patient groups, then we should accept that we have reached a ‘dead end’ and that further investigation is probably not warranted.

To perform the ‘extreme patient’ analysis, for each variable we prepared data sets (summarised in the final column of Table 2) comprising mass spectra for patients with the 20 and 30 highest and lowest values for each clinical variable of interest, yielding data sets containing 40 and 60 mass spectra, respectively. We note that the smaller data set provides better separation of the groups, but at the expense of containing fewer mass spectra for training. The population distributions for each clinical variable can be found in the SI.

In addition to the ‘all patient’ and ‘extreme patient’ data sets described above, two modifications were employed to investigate whether the performance of the machine learning models could be improved any further. In the first, we included clinically relevant demographic details that would be known at the point of treatment (the continuous parameters age, weight, height, body mass index (BMI), and body surface area (BSA), and categorical parameters of present/past smoking status, and presence of hypertension, diabetes, or previous cardiological history) in the training data set. Only patients for which a complete set of these parameters was available were included in this analysis, which reduced the data set sizes somewhat further. We chose not to include patient sex as a parameter when constructing the ‘extreme patient’ data sets due to the low number of female patients enrolled in the OxAMI study. The range for each continous clinical parameter was rescaled to the median intensity of the mass spectra before integration into the data set in order to avoid demographic parameters from dominating the machine learning process.

Finally, we employed feature reduction methods^52,53^ to construct data sets comprising a smaller number of *m*/*z* peaks. The feature reduction process identifies mass spectral peaks that correlate significantly with the clinical variables of interest. By training the machine learning algorithms using only these ‘most significant’ *m*/*z* peaks, we can potentially focus the machine learning process on the most relevant parts of the molecular fingerprints. The feature reduction process also reveals potential mass spectrometric biomarkers for clinical parameters of interest. Feature reduction was conducted on the training data within each 80 : 20 data partition prior to machine learning analysis. Three different approaches to feature reduction were explored:

(1) *Statistical tests*: the correlation coefficent between each peak and the clinical variable of interest was calculated. A parametric Pearson correlation coefficient test was used, as 98.9% of mass peaks were determined to be normally distributed by the Sharpio-Wilkes test with *P* < 0.05. Significant peaks (with *P* < 0.05) within the training data were then used as the input features, as opposed to the 990 *m*/*z* peaks used initially.

(2). *Analysis of overlap integrals for m*/*z peak intensity distributions*: for each clinical parameter, the normalised intensity distributions for each *m*/*z* peak were determined for the mass spectra assigned to the two defined patient groups. This yields two intensity distributions per *m*/*z* peak, corresponding to the ‘low’/‘high’ or ‘yes’/‘no’ patient groups for that parameter. If one calculates the overlap integral between these two intensity distributions, *m*/*z* for which there is little or no intensity variation between the two groups will return an integral close to one, and are likely to contain little information on the clinical parameter of interest, while those for which there is a significant intensity difference will return a significantly lower value, and are more likely to correlate with the clinical parameter under study. In this method of feature selection, the 40 peaks with the smallest overlap integrals were used to generate training data sets for the machine learning models.

(3) *Machine-learning-based feature ranking*: feature ranking analysis was conducted using the *χ*^2^ classification feature ranking method.^54^ The 40 features that were determined to have the highest ‘importance’ were used to generate training sets for the machine learning models.

The ability of each model to predict patient categories accurately was assessed though comparison of the % accuracy,1
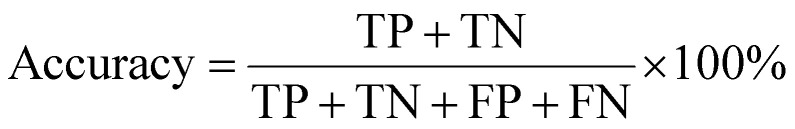
and Cohen's kappa score (*κ* score).^55^2
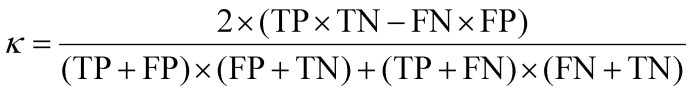
where TP, TN, FP, and FN are the number of true positives, true negatives, false positives, and false negatives, predicted by the model, respectively. The *κ* score varies between −1 and 1, and is interpreted in this study using the ‘levels of agreement’ as defined by McHugh and reproduced in Table 3.^55^ The mean and standard deviation of the values for the accuracy and *κ* score obtained across 200 partitionings of the data into test and training sets are reported. The values of *κ* that should be considered statistically sigificant (*p* < 0.05) depend on the number of samples.^56,57^ As a consequence, for small data sets, such as that used in the heart-failure analysis, and the ‘extreme patient’ data sets with *n* = 20 and *n* = 30, only scores of *κ* ≥ 0.6 and *κ* ≥ 0.5, respectively, are considered statistically significant.

**Table 3:** Interpretation of *κ* score in terms of levels of agreement, adapted from^55^

*κ* value	Level of agreement	Estimated equivalent % accuracy
<0.2	None	<60%
0.2–0.4	Weak	60–70%
0.4–0.6	Fair	70–80%
0.6–0.8	Moderate	80–90%
0.8–0.9	Strong	90–95%
>0.9	Very strong	>95%

## 3 Results and discussion

### 3.1 Prediction of clinical parameters

Tables 4 and 5 show the highest *κ* scores obtained for prediction of each of the clinical parameters (patient mortality, heart failure diagnosis, ischemic time, thrombus score, MVO, IMR, and troponin and creatinine levels) by the various trained machine learning models described in section 2.4. Table 4 shows the results for models trained on mass spectra from all available patients, while Table 5 shows results for models trained using mass spectra only for patients with the 20 or 30 highest and lowest values for each clinical variable. Full results tables for each model can be found in the SI.

**Table 4:** The highest *κ* scores achieved across all machine learning models when training the models on the complete set of patient data for each of the clinical variables of interest. The first three rows of the table define the training model setup (data set, clinical variables included or not included in the training input, and feature reduction method used), and the remaining rows report *κ* scores and their associated uncertainties for predictions of each clinical parameter. The highest score obtained for each variable is highlighted in green

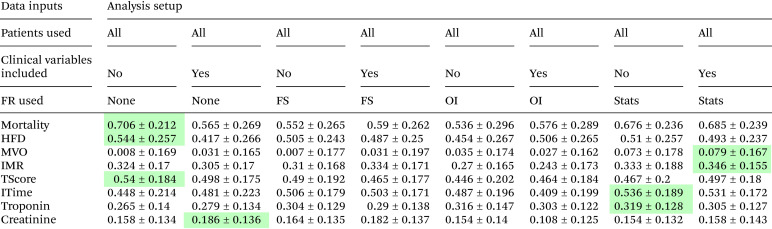

**Table 5:** As for Table 4, but for models trained on the ‘extreme patients’ data sets. The data set used in each case, named according to the relevant clinical variable (see Table 2) is listed in column 1

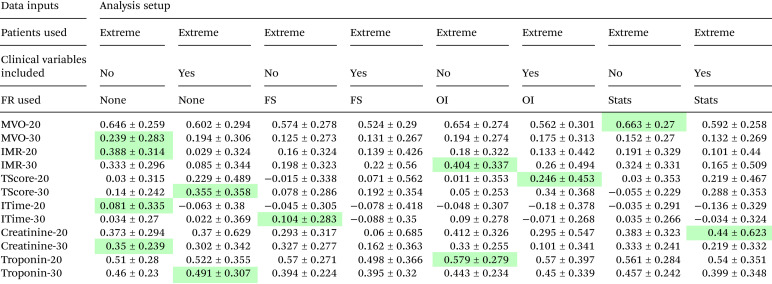

Before considering the results for each clinical parameter, we can make some general observations.

(1) For a given clinical parameter, the *κ* score, and therefore predictive power of the model, is reasonably consistent across all machine learning methods and feature reduction methods explored.

(2) Interestingly, reducing the data set so that only patients with the 20 or 30 highest and lowest values for the clinical parameter of interest were included led to a significant improvement in performance for prediction of MVO, creatinine, and troponin, but worse performance for prediction of IMR, ischemic time, and thrombus score.

(3) When comparing the performance of different feature reduction methods, we note that in the models trained on all patient data, the highest scores were isolated to data sets for which no feature reduction had been performed (for patient mortality, heart failure diagnosis, thrombus score, and creatinine), or for which feature reduction had been achieved *via* statistical analysis (for ischemic time, MVO, IMR, and troponin).

(4) Inclusion of clinical and demographic parameters within the training data did not improve the results in most cases.

(5) The uncertainties returned from the models were found to be largest in the prediction of patient mortality and heart failure diagnosis, consistent with the very small available data sets for these parameters. While *κ* scores for these analyses were statistically significant (*κ* > 0.6) in many cases, further studies with a larger number of samples are certainly required.

The best results achieved for each clinical variable across all models explored are summarised in Table 6. Despite the small data set, the most successful prediction was achieved for patient mortality (85% accuracy, *κ* 0.71), with classification of ‘high’ and ‘low’ MVO in extreme patient groups also predicted with a high accuracy of 83%, *κ* 0.66. Accuracies ranging from 70–80% were achieved for the remaining clinical parameters under investigation. The most successful machine learning models out of the five considered were LDA and SVM, which between them accounted for all of the best-performing models. LDA tends to perform well when the two classes are well represented by normal distributions, while SVM can cope more easily with outliers. Both models deal better with noisy and high-dimensionality data sets than the KNN and NBC models.

**Table 6:** The best results obtained for each categorical clinical variable, and the training data structure (data set, feature reduction method, and presence or absence of clinical and demographic input variables) required to achieve that result. Variables predicted with over 80% accuracy are highlighted green, variables predicted with over 70% accuracy are highlighted yellow

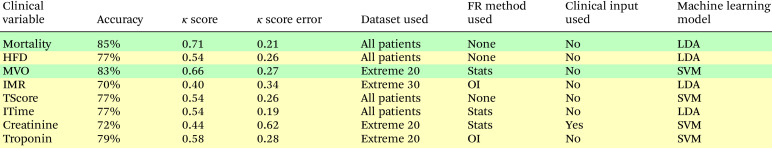

The high accuracies achieved for prediction of patient mortality and heart failure diagnosis after treatment for STEMI come with the caveat that since the number of patients available for the analysis was small (*n* = 46 and 50, with 23 and 25 patients per class, respectively), the accuracy may be somewhat exaggerated due to over-fitting. However, given the clinical importance of these predictions – the ability to identify patients at risk of fatality or serious complications post-STEMI at the point of presentation may enable such patients to be targeted with more aggressive treatments earlier on in the treatment pathway, potentially saving or prolonging life – further exploration is certainly warranted should samples become available.

Microvascular obstruction (MVO) is a known complication of pPCI in which the myocardial microcirculation remains obstructed despite reestablishment of blood flow through the affected coronary artery.^58^ In the OxAMI study, the extent of MVO is measured *via* a cardiac MRI scan at two days and six months after the STEMI. The majority of patients are measured to have either zero or very low MVO, reflecting successful treatment, and only a relatively small number have high values, reflecting a poor cardiac outcome. Given the threshold of zero for distinguishing between the ‘high MVO’ and ‘low MVO’ patient groups, and the fact that the patient distribution is highly skewed towards very low, near-zero, MVO values (see Fig. 1 of the SI), it is perhaps not surprising that machine learning models trained on the ‘all patient’ (*n* = 128) data were unable to assign patients correctly to these groups, even when feature reduction methods were employed. However, a drastic improvement was found when the models were trained on the ‘extreme patients’ data. A *κ* score of 0.66 and an accuracy of 83% was achieved with the smallest patient selection of *n* = 20 per class, reducing to *κ* = 0.24 and an accuracy of 62% in the larger *n* = 30 ‘extreme patients’ data set. These results imply that the machine learning models are capable of identifying patients at most risk of poor outcomes, opening up potential avenues for future investigation.

The index of microcirculatory resistance (IMR), measured *via* an invasive procedure while the patient is still catheterised, provides an assessment of cardiac microvascular function immediately post pPCI. IMR is often used as a marker for MVO, with values higher than 40 known to be associated with an adverse clinical outcome. However, in around a third of cases the two parameters can be surprisingly dicordant,^59^ perhaps due to resolution of reversible symptoms associated with edema relatively early on in the course of the patient's recovery.^60^ Given the success of our machine learning approach at identifying patients with high MVO, it is perhaps surprising that there was very little success in predicting IMR. Using the ‘all patients’ data set (*n* = 126), the trained models all performed equally poorly, achieving no-to-low agreement, with only a slight improvement to a best accuracy of 67% (*κ* = 0.346, low agreement) when feature selection was implemented. When using the ‘extreme patients’ data the performance of the different models was more varied, with the best result (70% accuracy, *κ* = 0.40) obtained for the SVM model trained on the IMR-30 data set with overlap integral feature selection.

Ischemic time was predicted with low accuracy using the ‘all patients’ (*n* = 92) data set, improving to fair agreement (77% accuracy, *κ* = 0.54) when feature selection was implemented. Limiting the data set to ‘extreme value’ patients led to a drastic reduction in predictive power to no better than a random guess, implying that a larger data set is beneficial when characterising this parameter. Since ischemic time is often relatively subjective, and usually known at the time of patient admission, the ability to ‘predict’ this quantity is probably not useful in developing diagnostic or prognostic indicators for heart health following STEMI. However, our results do show that there are detectable metabolic changes in plasma that correlate with ischemic time, which with further investigation may be beneficial in understanding physiological changes associated with coronary artery occlusion.

Thrombus score (TScore) or thrombus burden is measured during pPCI by passage of a flow guide wire, and measures the extent of occlusion to the culprit coronary artery. It is defined on a scale from 0 to 5 in terms of partial/total radial blockage of the artery and extent of the blockage along the vessel length.^13,61,62^ The performance of the machine learning models in relation to TScore was very similar to that for ischemic time, showing fair (77%) agreement (*κ* = 0.54) when trained on the ‘all patients’ data, and no predictive power when trained only on the ‘extreme patients’ data. The ability to predict thrombus score implies that thrombus size has a measurable effect on the metabolic profile of the patient that could potentially prove useful in making clinical decisions.

The final two clinical parameters, peak troponin and creatinine levels, are somewhat different from the other parameters in that they relate to concentrations of specific identifiable chemical species. Both are biomarkers for STEMI, with troponin being the more sensitive and specific, and therefore more widely used in clinical practice.

Despite the fact that creatinine is a small molecule lying within the *m*/*z* range of our mass spectrometer, none of the machine learning models performed particularly well in classifying patients to ‘high’ or ‘low’ groups. No model performed significantly better than a random guess when presented with the data set for all 242 patient samples, and only ‘low’ predictive accuracy was obtained when the data set was limited to the 20 patients with the highest and lowest values for creatinine. The best performance was found when feature reduction was employed and demographic variables were included, using the SVM model, but this only resulted in a *κ* score of 0.44 and an accuracy of 72%. Creatinine will be discussed further in section 3.2 when considering the *m*/*z* peaks of interest revealed through the feature reduction process.

Troponin is a protein with a molecular mass outside of the range of our mass spectrometer; hence plasma troponin cannot be measured directly in the present study, but we may expect to be sensitive to changes in the plasma metabolome resulting from alterations in biological pathways involving troponin. Using the ‘all patients’ (*n* = 220) data set the best result was an accuracy of 66% (*κ* = 0.319), with similar results across all machine learning models trained on the feature-selected data. Using the data set comprising the patients with the 20 highest and lowest troponin values improved the accuracy to 78% and *κ* to 0.58, with the best results for the SVM model. Performance was fairly consistent across all models apart from RFC, which performed poorly in all cases.

### 3.2 Identification of potential biomarkers by feature reduction

**Fig. 3:**
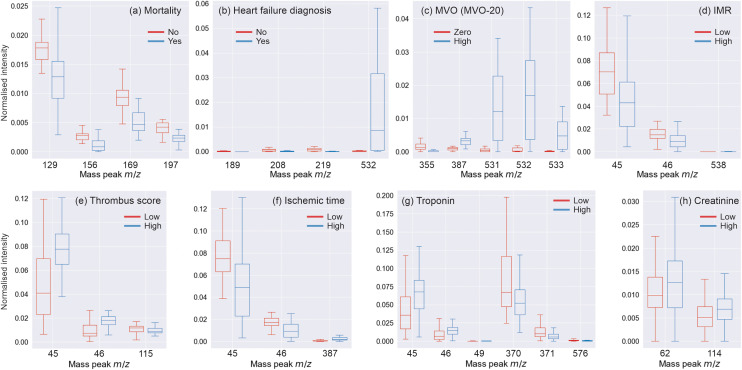
Box and whisker plots showing the distribution of normalised peak intensities within the ‘all patients’ data set (except for MVO, which used the MVO-20 data set) for *m*/*z* peaks identified by the feature reduction analysis as being of interest for each patient group and clinical parameter investigated: (a) patient mortality; (b) heart failure diagnosis (inset shows the same data on a magnified scale); (c) MVO (MVO-20 data set); (d) IMR; (e) thrombus score; (f) ischemic time; (g) troponin; (h) creatinine. In each plot, the central line represents the data median, with the inner box representing the inter-quartile range between the first and third quartiles. The whiskers extend from the box to the farthest data point lying within 1.5 × the inter-quartile range from the box. Significance was measured by an independent *t*-test.

Interrogating the results of the various feature reduction methods allows us to identify which *m*/*z* peaks are most important in distinguishing between the patient groups of interest. There was generally found to be some overlap between the peaks identified by the three different feature reduction methods, but there were also substantial differences. In a first attempt to identify potential mass spectrometric biomarkers associated with each clinical variable, we applied the (admittedly somewhat arbitrary) criterion that qualifying *m*/*z* peaks should be identified in more than 75% of the data training partitions for all three feature reduction methods employed. Table 7 shows the results of this analysis, which returned between one and six *m*/*z* peaks of interest for each clinical variable. Fig. 3 shows the intensity distributions for these peaks within the different patient groups associated with each clinical parameter, in the form of box and whisker plots. The *t* statistic and *p* score determined for each *m*/*z* peak can be found in Table 20 of the SI.

**Table 7:** The *m*/*z* ion peaks identified as being important for classification of patients according to each clinical parameter by all three of the feature reduction methods employed

Clinical variable	*m*/*z* peaks (all patients)	*m*/*z* peaks (extreme patient groups)
Mortality	129, 169, 156, 197	NA
HFD	208, 219, 532, 189	NA
MVO	None	355, 387, 531, 532, 533
IMR	45, 46	538
TScore	45, 46	115
ITime	45, 46, 387	None
Troponin	45, 46, 370, 371	45, 46, 49, 576
Creatinine	114	62, 114

The present study is focused on a pattern recognition approach, and the resolution of our ASAP-MS instrument is not high enough for us to provide a definitive identification of the molecules giving rise to these peaks. While metabolite databases such as the Human Metabolome Database (HMDB)^63,64^ can identify some potential candidates, more sophisticated methods such as LC-MS will be needed in order to progress work on identifying molecules of interest. However, with this caveat in place, there are a few masses worth commenting on.

The pair of *m*/*z* values 45 and 46 were found to be important for four of the clinical parameters – ischemic time, IMR, thrombus score, and troponin – always as a pair. The ASAP-MS instrument tends to generate protonated positive ions, so assuming a protonated parent ion, potential molecular candidates are CO_2_ (carbon dioxide), C_2_H_4_O (acetaldehyde), and C_2_H_7_N (dimethylamine or ethylamine). All of these molecules are common metabolites relevant to numerous anabolic and catabolic pathways that are relevant in many disease states. Alternatively, the *m*/*z* 45 and 46 peaks may be fragment ions generated from larger metabolites.

Two peaks were found to be important for successful classification of creatinine, one of which was at *m*/*z* 114. As creatinine has a molecular mass of 113.12 g mol^−1^, it is highly likely that this peak corresponds to the protonated parent ion. However, the accuracy with which creatinine is predicted from the mass spectra is low, and the intensity of this peak shows no correlation with the clinically reported value for creatinine. There are a number of possible reasons for this discrepancy. The peak creatinine level measured in the clinic is recorded at a different time from the plasma sample collected from the coronary artery during pPCI. The rate of creatinine excretion *via* the kidneys differs significantly between patients,^65,66^ leading to considerable inter-patient variability in the correlation between the two creatinine levels. It is also possible that blood plasma that has been stagnating in the region occluded by the coronary thrombus is not representative of the systemic blood creatinine levels. Finally, mass spectrometric analysis is only semi-quantitative, in comparison with the highly quantitative clinical measurement of creatinine.^67,68^

Table 8 shows the results of training the machine learning models using only the very small number of mass peaks identified in Table 7, compared against the best results obtained using all *m*/*z* peaks. We see that for the most part the accuracies are similar in the two analyses, implying that the peaks found by the feature reduction algorithms account for much of the variation between the patient groups of interest. For creatinine, HFD, troponin, and IMR, the *κ* scores obtained using the feature-selected *m*/*z* peaks were slightly higher than those obtained using all peaks, while for mortality, thrombus score, ischemic time, and MVO the *κ* score was lower, though still in the ‘fair agreement’ range. The fact that a very small number of *m*/*z* peaks seem to be of high importance when classifying patients is a positive outcome for any future clinical applications, since focusing on quantification of a small number of molecules is likely to improve the efficiency of any analysis considerably.

**Table 8:** The *κ* scores obtained when the machine learning models were trained using only the *m*/*z* peaks found to be important for classification (see Table 7) compared against the best result found using all *m*/*z* peaks. Changes in *κ* score of less than or greater than 0.05 are highlighted in red and green respectively

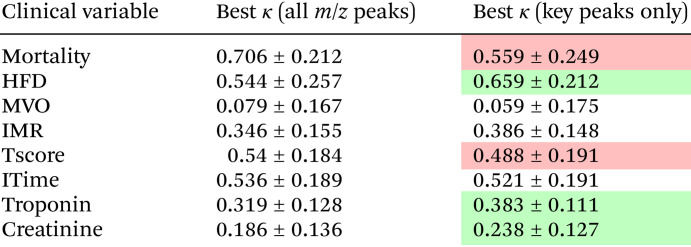

## 4 Conclusions and outlook

We have presented results from a proof-of-concept investigation into the use of a combined ASAP-MS and machine learning analysis of blood plasma for the prediction of clinically relevant pathologies and outcomes for patients participating in the OxAMI study into ST-segment elevation myocardial infarction.

Prediction of the nine clinical variables was completed through analysis of ASAP-MS measurements of coronary aspirate plasma, with patient mortality and MVO predicted with over 80% accuracy; HFD, ITime, peak troponin and TScore predicted with over 76% accuracy; and creatinine and IMR predicted with over 70% accuracy. Feature reduction methods were applied to try to improve the speed and accuracy of the models used, but no single method was found to give large improvements to model accuracy. It was found that the analysis set-up requirements were specific to the variable being analysed. Using the feature reduction methods, peaks found to be important for classification were identified. The biomolecules responsible for these peaks will be investigated in further studies, with the aim of learning more about the pathology of STEMI patients and potentially developing clinical tools for identifying high risk STEMI patients.

The patient numbers used in this analysis were low, which may contribute to both over fitting by machine learning models, or reduced accuracy due to sample representation. Ideally, this study will now be repeated with larger patient numbers, potentially from a number of studies similar in set-up to the OxAMI study. Analysis of coronary aspirate plasma was completed in this early investigation as this resource was not deemed to be as valuable to the biological database as other samples. As discussed previously, coronary aspirate blood has been in some cases sat stagnant in the occluded coronary artery for unknown periods of time, and will be contaminated to differing degrees with saline solution and medication administered to aid the PPCI procedure. The reproducibility in terms of composition, consistency, and representation of the patient for coronary aspirate blood is likely to be low compared to plasma samples taken from a standard blood draw. This increased variation will have a large impact on the results obtained using ASAP-MS. The authors have found in other work that conducting ASAP-MS on venous blood plasma is noticeably more reproducible within a similar dataset (results to be published). The results of this study have determined that this method can achieve impressive positive results that may help to develop new clinical tools for the assessment of patient risk. The analysis will now be repeated on a range of biological samples that are likely more consistent that coronary aspirate samples, such as venous blood samples and coronary sinus plasma.

## Author contributions

The study was conceived by CV and ASJEB with support from GLDM, RK, and KC. The ASAP-MS and data analysis methodology was developed by ASJEB, TM, and CV. Experiments were performed by ASJEB and TM, software was written and data analysis performed by ASJEB. Samples were provided by the OxAMI study, GLDM, RK, and KC. Funding was obtained by CV, KC, and GLDM. Supervision was provided by CV and GLDM. ASJEB and CV wrote the original draft, and all authors were involved in review and editing.

## Conflicts of interest

The authors have no competing interests to declare.

## Ethics statement

The authors confirm that the work described has been carried out in accordance with The Code of Ethics of the World Medical Association (Declaration of Helsinki) for experiments involving humans. Institutional Review Board (IRB)/Ethics Committee approval was obtained from the Oxford Regional Ethics Committee.

## Abbreviations

ASAP-MS: Atmospheric solids analysis probe mass spectrometry
BMI: Body mass index
CPU: Central processing unit
HFD: Heart failure diagnosis
IMR: Index of microcirculatory resistance
ITime: Ischemic time
KNN: K nearest neighbours
LDA: Linear discriminant analysis
MI: Myocardial infarction
MVO: Microvascular obstruction
NBC: Naïve Bayes classifier
NSTEMI: Non-ST-segment-elevation myocardial infarction
OxAMI: Oxford acute myocardial infarction study
pPCI: Primary percutaneous coronary intervention
RFC: Random forest classifier
STEMI: ST-segment-elevation myocardial infarction
SVM: Support vector machines
TScore: Thrombus score

## Supplementary Material

AN-150-D5AN00565E-s001

AN-150-D5AN00565E-s002

AN-150-D5AN00565E-s003

AN-150-D5AN00565E-s004

AN-150-D5AN00565E-s005

AN-150-D5AN00565E-s006

AN-150-D5AN00565E-s007

## Data Availability

Further data are available in the Supplementary Information (SI). This contains patient population distributions in relation to the clinical parameters of interest, further information about the machine learning methods employed in the data analysis, data tables summarising the full set of machine-learning outcomes, summaries of the best performing methods for each analysis, and a list of *m*/*z* peaks identified using feature reduction methods. The Supplementary information is available at DOI: https://doi.org/10.1039/d5an00565e. Any data not included in the SI are available on request from the corresponding author.

## References

[cit1] Doost Hosseiny A., Moloi S., Chandrasekhar J., Farshid A. (2016). Mortality pattern and cause of death in a long-term follow-up of patients with STEMI treated with primary PCI. Open Heart.

[cit2] Weston C. , Perwaiz S., Wang J., Kerr J., Dana A., de Belder M., Ludman P., Mamas M., Gale C., Wragg A., Miles C., Alkofer B., Keys A., Ighofose C., Quinn T. and Goodfellow J., Management of Heart Attack: analyses from the Myocardial Ischaemia National Audit Project (MINAP) and the National Audit of Percutaneous Coronary Intervention (NAPCI), Myocardial ischaemia/minap (heart attack audit) report, 2023

[cit3] Karam N., Bataille S., Marijon E., Tafflet M., Benamer H., Caussin C., Garot P., Juliard J., Pires V., Boche T., Dupas F., Le Bail G., Lamhaut L., Simon B., Allonneau A., Mapouata M., Loyeau A., Empana J., Lapostolle F., Spaulding C., Jouven X., Lambert Y. (2019). Incidence, Mortality, and Outcome-Predictors of Sudden Cardiac Arrest Complicating Myocardial Infarction Prior to Hospital Admission. Circ.: Cardiovasc. Interventions.

[cit4] Krittanawong C., Yue B., Mahtta D., Narasimhan B., Kumar A., Wang Z., Sharma S. K., Tamis-Holland J. E., Brar S. S., Mehran R., Alam M., Jneid H., Virani S. S. (2022). Readmission in Patients With ST-Elevation Myocardial Infarction in 4 Age Groups (¡45, ¿45 to ¡60, 60 to ¡75, and ¿75). Am. J. Cardiol..

[cit5] Kwok C. S., Narain A., Pacha H. M., Lo T. S., Holroyd E. W., Alraies M. C., Nolan J., Mamas M. A. (2020). Readmissions to Hospital After Percutaneous Coronary Intervention: A Systematic Review and Meta-Analysis of Factors Associated with Readmissions. Cardiovasc. Revasc. Med..

[cit6] Rymer J. A., Chen A. Y., Thomas L., Fonarow G. C., Peterson E. D., Wang T. Y. (2019). Readmissions After Acute Myocardial Infarction: How Often Do Patients Return to the Discharging Hospital?. J. Am. Heart Assoc..

[cit7] Sritharan H. P., Nguyen H., Ciofani J., Bhindi R., Allahwala U. K. (2024). Machine-learning based risk prediction of in-hospital outcomes following STEMI: the STEMI-ML score. Front. Cardiovasc. Med..

[cit8] Kasim S., Amir Rudin P. N. F., Malek S., Ibrahim K. S., Wan Ahmad W. A., Fong A. Y. Y., Lin W. Y., Aziz F., Ibrahim N. (2024). Ensemble machine learning for predicting in-hospital mortality in Asian women with ST-elevation myocardial infarction (STEMI). Sci. Rep..

[cit9] Wang C. H., Wang H. T., Wu K. H., Cheng F. J., Cheng C. I., Kung C. T., Chen F. C. (2022). Comparison of Different Risk Scores for Prediction of In-Hospital Mortality in STEMI Patients Treated with PPCI. Emerg. Med. Int..

[cit10] Shakhgeldyan K. I., Kuksin N. S., Domzhalov I. G., Rublev V. Y., Geltser B. I. (2024). Interpretable machine learning for in-hospital mortality risk prediction in patients with ST-elevation myocardial infarction after percutaneous coronary interventions. Comput. Biol. Med..

[cit11] Chen P., Wang B., Zhao L., Ma S., Wang Y., Zhu Y., Zeng X., Bai Z., Shi B. (2023). Machine learning for predicting intrahospital mortality in ST-elevation myocardial infarction patients with type 2 diabetes mellitus. BMC Cardiovasc. Disord..

[cit12] Kasim S., Malek S., Aziz M. F., Ibrahim K. S. (2022). Machine learning to predict in-hospital mortality risk among heterogeneous STEMI patients with diabetes. Eur. Heart J..

[cit13] Montalto C., Kotronias R. A., Marin F., Terentes-Printzios D., Shanmuganathan M., Emfietzoglou M., Scalamera R., Porto I., Langrish J., Lucking A., Choudhury R., Kharbanda R., Channon K. M., De Maria G. L., Banning A. (2021). Pre-procedural ATI score (age-thrombus burden-index of microcirculatory resistance) predicts long-term clinical outcomes in patients with ST elevation myocardial infarction treated with primary percutaneous coronary intervention. Int. J. Cardiol..

[cit14] Scarsini R., Shanmuganathan M., De Maria G. L., Borlotti A., Kotronias R. A., Burrage M. K., Terentes-Printzios D., Langrish J., Lucking A., Fahrni G., Cuculi F., Ribichini F., Choudhury R. P., Kharbanda R., Ferreira V. M., Channon K. M., Banning A. P. (2021). Coronary Microvascular Dysfunction Assessed by Pressure Wire and CMR After STEMI Predicts Long-Term Outcomes. JACC: Cardiovasc. Imaging.

[cit15] Waard G. A., Fahrni G., De Wit D., Kitabata H., Williams R., Patel N., Teunissen P. F., Van De Ven P. M., Umman S., Knaapen P., Perera D., Akasaka T., Sezer M., Kharbanda R. K., Van Royen N. (2018). Hyperaemic microvascular resistance predicts clinical outcome and microvascular injury after myocardial infarction. Heart.

[cit16] Fahrni G., Wolfrum M., De Maria G. L., Cuculi F., Dawkins S., Alkhalil M., Patel N., Forfar J. C., Prendergast B. D., Choudhury R. P., Channon K. M., Banning A. P., Kharbanda R. K. (2017). Index of Microcirculatory Resistance at the Time of Primary Percutaneous Coronary Intervention Predicts Early Cardiac Complications: Insights From the OxAMI (Oxford Study in Acute Myocardial Infarction) Cohort. J. Am. Heart Assoc..

[cit17] Patel N., Petraco R., Dall'Armellina E., Kassimis G., De Maria G. L., Dawkins S., Lee R., Prendergast B. D., Choudhury R. P., Forfar J. C., Channon K. M., Davies J., Banning A. P., Kharbanda R. K. (2015). Zero-Flow Pressure Measured Immediately After Primary Percutaneous Coronary Intervention for ST-Segment Elevation Myocardial Infarction Provides the Best Invasive Index for Predicting the Extent of Myocardial Infarction at 6 Months: An OxAMI Study (Oxford Acute Myocardial Infarction). JACC: Cardiovasc. Interv..

[cit18] Scarsini R., De Maria G. L., Borlotti A., Kotronias R. A., Langrish J. P., Lucking A. J., Choudhury R. P., Ferreira V. M., Ribichini F., Channon K. M., Kharbanda R. K., Banning A. P. (2019). Incremental Value of Coronary Microcirculation Resistive Reserve Ratio in Predicting the Extent of Myocardial Infarction in Patients with STEMI. Insights from the Oxford Acute Myocardial Infarction (OxAMI) Study. Cardiovasc. Revasc. Med..

[cit19] De Maria G. L., Alkhalil M., Wolfrum M., Fahrni G., Borlotti A., Gaughran L., Dawkins S., Langrish J. P., Lucking A. J., Choudhury R. P., Porto I., Crea F., Dall'Armellina E., Channon K. M., Kharbanda R. K., Banning A. P. (2017). The ATI score (age-thrombus burden-index of microcirculatory resistance) determined during primary percutaneous coronary intervention predicts final infarct size in patients with ST-elevation myocardial infarction: a cardiac magnetic resonance validation study. EuroIntervention.

[cit20] De Maria G. L., Fahrni G., Alkhalil M., Cuculi F., Dawkins S., Wolfrum M., Choudhury R. P., Forfar J. C., Prendergast B. D., Yetgin T., Van Geuns R. J., Tebaldi M., Channon K. M., Kharbanda R. K., Rothwell P. M., Valgimigli M., Banning A. P. (2016). A tool for predicting the outcome of reperfusion in ST-elevation myocardial infarction using age, thrombotic burden and index of microcirculatory resistance (ATI score). EuroIntervention.

[cit21] Rankin-Turner S., Reynolds J. C., Turner M. A., Heaney L. M. (2022). Applications of ambient ionization mass spectrometry in 2021: An annual review. Anal. Sci. Adv..

[cit22] Li L. H., Hsieh H. Y., Hsu C. C. (2017). Clinical Application of Ambient Ionization Mass Spectrometry. Mass Spectrom..

[cit23] Wiseman J. M., Evans C. A., Bowen C. L., Kennedy J. H. (2010). Direct analysis of dried blood spots utilizing desorption electrospray ionization (DESI) mass spectrometry. Analyst.

[cit24] Van Hese L., De Vleeschouwer S., Theys T., Larivière E., Solie L., Sciot R., Siegel T. P., Rex S., Heeren R. M. A., Cuypers E. (2022). Towards real-time intraoperative tissue interrogation for REIMS-guided glioma surgery. J. Mass Spectrom. Adv. Clin. Lab.

[cit25] Crevelin E. J., Salami F. H., Alves M. N. R., De Martinis B. S., Crotti A. E. M., Moraes L. A. B. (2016). Direct Analysis of Amphetamine Stimulants in a Whole Urine Sample by Atmospheric Solids Analysis Probe Tandem Mass Spectrometry. J. Am. Soc. Mass Spectrom..

[cit26] Arrizabalaga-Larrañaga A., Zoontjes P. W., Lasaroms J. J. P., Nielen M. W. F., Blokland M. H. (2022). Simplified screening approach of anabolic steroid esters using a compact atmospheric solid analysis probe mass spectrometric system. Anal. Bioanal. Chem..

[cit27] Carrizo D., Nerín I., Domeño C., Alfaro P., Nerín C. (2016). Direct screening of tobacco indicators in urine and saliva by Atmospheric Pressure Solid Analysis Probe coupled to quadrupole-time of flight mass spectrometry (ASAP-MS-Q-TOF-). J. Pharm. Biomed. Anal..

[cit28] Tian H., Zhao X., Zhang Y., Xia Z. (2023). Abnormalities of glucose and lipid metabolism in myocardial ischemia-reperfusion injury. Biomed. Pharmacother..

[cit29] Beck A. G., Muhoberac M., Randolph C. E., Beveridge C. H., Wijewardhane P. R., Kenttämaa H. I., Chopra G. (2024). Recent Developments in Machine Learning for Mass Spectrometry. ACS Meas. Sci. Au.

[cit30] Verbeeck N., Caprioli R. M., Van de Plas R. (2020). Unsupervised machine learning for exploratory data analysis in imaging mass spectrometry. Mass Spectrom. Rev..

[cit31] Tran N. H., Qiao R., Xin L., Chen X., Liu C., Zhang X., Shan B., Ghodsi A., Li M. (2018). Deep learning enables de novo peptide sequencing from data-independent-acquisition mass spectrometry. Nat. Methods.

[cit32] Abdelmoula W. M., Stopka S. A., Randall E. C., Regan M., Agar J. N., Sarkaria J. N., Wells W. M., Kapur T., Agar N. Y. R. (2022). massNet: integrated processing and classification of spatially resolved mass spectrometry data using deep learning for rapid tumor delineation. Bioinformatics.

[cit33] OXAMI: Oxford Acute Myocardial Infarction Study website: https://oxami.org.uk/

[cit34] Cuculi F., Herring N., De Caterina A. R., Banning A. P., Prendergast B. D., Forfar J. C., Choudhury R. P., Channon K. M., Kharbanda R. K. (2013). Relationship of plasma neuropeptide Y with angiographic, electrocardiographic and coronary physiology indices of reperfusion during ST elevation myocardial infarction. Heart.

[cit35] Pham T. D., Wang H., Zhou X., Beck D., Brandl M., Hoehn G., Azok J., Brennan M.-L., Hazen S. L., Li K., Wong S. T. C. (2008). Computational prediction models for early detection of risk of cardiovascular events using mass spectrometry data. IEEE Trans. Inf. Technol. Biomed..

[cit36] Hoogeveen R. M., Pereira J. P. B., Nurmohamed N. S., Zampoleri V., Bom M. J., Baragetti A., Boekholdt S. M., Knaapen P., Khaw K.-T., Wareham N. J., Groen A. K., Catapano A. L., Koenig W., Levin E., Stroes E. S. G. (2020). Improved cardiovascular risk prediction using targeted plasma proteomics in primary prevention. Eur. Heart J..

[cit37] Ottosson F., Engström G., Orho-Melander M., Melander O., Nilsson P. M., Johansson M. (2024). Plasma metabolome predicts aortic stiffness and future risk of coronary artery disease and mortality after 23 years of follow-up in the general population. J. Am. Heart Assoc..

[cit38] Hjort M., Eggers K. M., Lindhagen L., Baron T., Erlinge D., Jernberg T., Marko-Varga G., Rezeli M., Spaak J., Lindahl B. (2021). Differences in biomarker concentrations and predictions of long-term outcome in patients with ST-elevation and non-ST-elevation myocardial infarction. Clin. Biochem..

[cit39] Shavadia J. S., Alemayehu W., deFilippi C., Westerhout C. M., Tromp J., Granger C. B., Armstrong P. W., Van Diepen S. (2022). Novel multi-marker proteomics in phenotypically matched patients with ST-segment myocardial infarction: association with clinical outcomes. J. Thromb. Thrombolysis.

[cit40] Borlotti A., Jerosch-Herold M., Liu D., Viliani D., Bracco A., Alkhalil M., De Maria G. L., Channon K. M., Banning A. P., Choudhury R. P., Neubauer S., Kharbanda R. K., Dall-Armellina E. (2019). Acute Microvascular Impairment Post-Reperfused STEMI Is Reversible and Has Additional Clinical Predictive Value: A CMR OxAMI Study. JACC: Cardiovasc. Imaging.

[cit41] Cuculi F., Herring N., De Caterina A. R., Banning A. P., Prendergast B. D., Forfar J. C., Choudhury R. P., Channon K. M., Kharbanda R. K. (2013). Relationship of plasma neuropeptide Y with angiographic, electrocardiographic and coronary physiology indices of reperfusion during ST elevation myocardial infarction. Heart.

[cit42] De Maria G. L., Cuculi F., Patel N., Dawkins S., Fahrni G., Kassimis G., Choudhury R. P., Forfar J. C., Prendergast B. D., Channon K. M., Kharbanda R. K., Banning A. P. (2015). How does coronary stent implantation impact on the status of the microcirculation during primary percutaneous coronary intervention in patients with ST-elevation myocardial infarction?. Eur. Heart J..

[cit43] Eardley-Brunt A. S. J., Jones A., Mills T., Song L., Kotronias R., Lapolla P., Handa A., Lee R., Channon K., de Maria G. L. (2025). Development of an optimised method for the analysis of human blood plasma samples by atmospheric solids analysis probe mass spectrometry. Int. J. Mass Spectrom..

[cit44] Song L., Reese J. G., Platt M. A., Lewis C., Eardley-Brunt A. S. J., Sun B., Ansorge O., Vallance C. (2025). Advancing atmospheric solids analysis probe mass spectrometry applications: a multifaceted approach to optimising clinical data set generation. Analyst.

[cit45] Eardley-Brunt A. S. J., Song L., study The OxAMI, Vallance C. (2025). Optimising the choice of normalisation method for use in machine-learning classification of human blood plasma ambient ionisation mass spectra. Int. J. Mass Spectrometry.

[cit46] Fahrni G., Wolfrum M., De Maria G. L., Cuculi F., Dawkins S., Alkhalil M., Patel N., Forfar J. C., Prendergast B. D., Choudhury R. P., Channon K. M., Banning A. P., Kharbanda R. K. (2017). Index of Microcirculatory Resistance at the Time of Primary Percutaneous Coronary Intervention Predicts Early Cardiac Complications: Insights From the OxAMI (Oxford Study in Acute Myocardial Infarction) Cohort. J. Am. Heart Assoc..

[cit47] Scarsini R., Shanmuganathan M., De Maria G. L., Borlotti A., Kotronias R. A., Burrage M. K., Terentes-Printzios D., Langrish J., Lucking A., Fahrni G., Cuculi F., Ribichini F., Choudhury R. P., Kharbanda R., Ferreira V. M., Channon K. M., Banning A. P. (2021). Coronary Microvascular Dysfunction Assessed by Pressure Wire and CMR After STEMI Predicts Long-Term Outcomes. JACC: Cardiovasc. Imaging.

[cit48] Guo G., Wang H., Bell D., Bi Y., Greer K. (2003). KNN Model-Based Approach in Classification. Lect. Notes Comput. Sci..

[cit49] Cortes C., Vapnik V., Saitta L. (1995). Support-vector networks. Mach. Learn..

[cit50] Zhou Z. , Machine Learning, Springer, Singapore, 2021

[cit51] Ho T. K.

[cit52] Kira K. and Rendell L. A.

[cit53] Spencer R., Thabtah F., Abdelhamid N., Thompson M. (2020). Exploring feature selection and classification methods for predicting heart disease. Digital Health.

[cit54] Liu H. and Setiono R.

[cit55] McHugh M. L. (2012). Interrater reliability: the kappa statistic. Biochem. Med..

[cit56] Donner A., Eliasziw M. (1992). A goodness-of-fit approach to inference procedures for the kappa statistic: confidence interval construction, significance testing, and sample size estimation. Stat. Med..

[cit57] Sim J., Wright C. C. (2005). The kappa statistic in reliability studies: use, interpretation, and sample size requirements. Phys. Ther..

[cit58] Abbas A., Matthews G. H., Brown I. W., Shambrook J. S., Peebles C. R., Harden S. P. (2015). Cardiac MR assessment of microvascular obstruction. Br. J. Radiol..

[cit59] de Maria G. L., Alkhalil M., Wolfrum M., Fahrni G., Borlotti A., Gaughran L., Dawkins S., Langrish J. P., Lucking A. J., Choudhury R. P., Porto I., Crea F., Dall'Armellina E., Channon K. M., Kharbanda R. K., Banning A. P. (2019). Index of microcirculatory resistance as a tool to characterize microvascular obstruction and to predict infarct size regression in patients with STEMI undergoing primary PCI. JACC: Cardiovasc. Imaging.

[cit60] Bulluck H., Berry C. (2019). Toward improving our understanding of the relationship between IMR and MVO in STEMI patients. JACC: Cardiovasc. Imaging.

[cit61] Sianos G., Papafaklis M. I., Daemen J., Vaina S., Van Mieghem C. A., Van Domburg R. T., Michalis L. K., Serruys P. W. (2007). Angiographic stent thrombosis after routine use of drug-eluting stents in ST-segment elevation myocardial infarction: the importance of thrombus burden. J. Am. Coll. Cardiol..

[cit62] Gibson C. M., De Lemos J. A., Murphy S. A., Marble S. J., McCabe C. H., Cannon C. P., Antman E. M., Braunwald E. (2001). Combination therapy with abciximab reduces angiographically evident thrombus in acute myocardial infarction: a TIMI 14 substudy. Circulation.

[cit63] Wishart D. S., Tzur D., Knox C., Eisner R., Guo A. C., Young N., Cheng D., Jewell K., Arndt D., Sawhney S., Fung C., Nikolai L., Lewis M., Coutouly M.-A., Forsythe I., Tang P., Shrivastava S., Jeroncic K., Stothard P., Amegbey G., Block D., Hau D. D., Wagner J., Miniaci J., Clements M., Gebremedhin M., Guo N., Zhang Y., Duggan G. E., MacInnis G. D., Weljie A. M., Dowlatabadi R., Bamforth F., Clive D., Greiner R., Li L., Marrie T., Sykes B. D., Vogel H. J., Querengesser L. (2007). HMDB - the Human Metabolome Database. Nucleic Acids Res..

[cit64] Wishart D. S., Guo A., Oler E., Wang F., Anjum A., Peters H., Dizon R., Sayeeda Z., Tian S., Lee B. L., Berjanskii M., Mah R., Yamamoto M., Jovel J., Torres-Calzada C., Hiebert-Giesbrecht M., Lui V. W., Varshavi D., Allen D., Arndt D., Khetarpal N., Sivakumaran A., Harford K., Sanford S., Yee K., Cao X., Budinski Z., Liigand J., Zhang L., Zheng J., Mandal R., Karu N., Dambrova M., Schiöth H. B., Greiner R., Gautam V. (2022). HMDB 5.0 - the Human Metabolome Database for 2022. Nucleic Acids Res..

[cit65] Hahn T., Yao S., Dunford L. M., Thomas J., Lohr J., Arora P., Battiwalla M., Smiley S. L., McCarthy P. L. (2009). A Comparison of Measured Creatinine Clearance versus Calculated Glomerular Filtration Rate for Assessment of Renal Function before Autologous and Allogeneic BMT. Biol. Blood Marrow Transplant..

[cit66] Measurement of kidney function, UK Kidney website: https://www.ukkidney.org/health-professionals/information-resources/uk-eckd-guide/measurement-kidney-function

[cit67] van den Berg R. A., Hoefsloot H. C., Westerhuis J. A., Smilde A. K., van der Werf M. J. (2006). Centering, scaling, and transformations: improving the biological information content of metabolomics data. BMC Genomics.

[cit68] Misra B. B. (2020). Data normalization strategies in metabolomics: Current challenges, approaches, and tools. Eur. J. Mass Spectrom..

