# Supplementary figures and images for: Prediction of clinical outcomes of ST-elevated myocardial infarction patients using atmospheric solids analysis probe mass spectrometry and machine learning
Source: Analyst. 2025 Sep 24;150(22):4982–96. doi: 10.1039/d5an00565e (PMC12498272; doi:10.1039/d5an00565e)

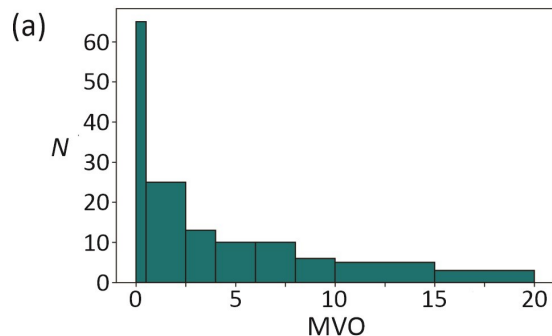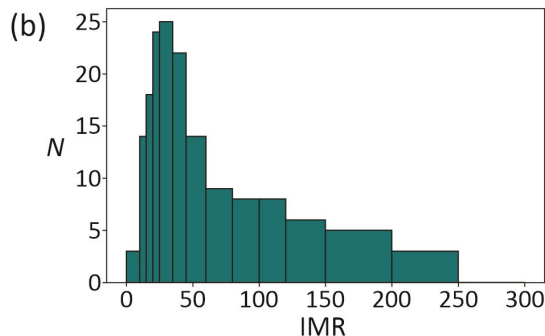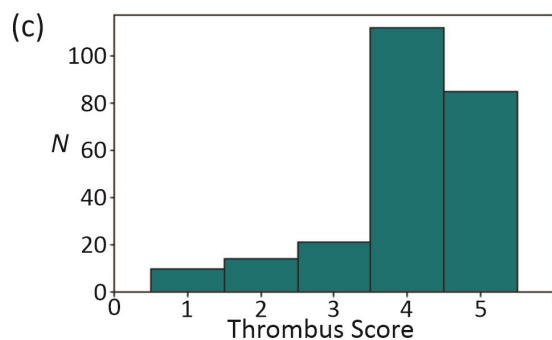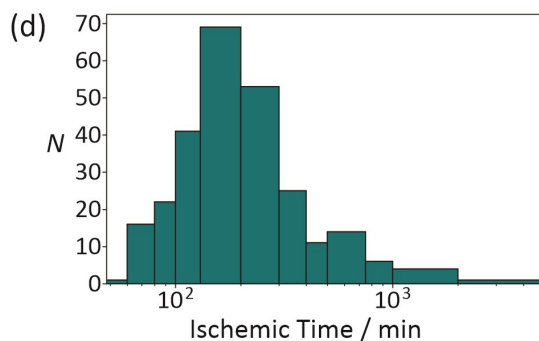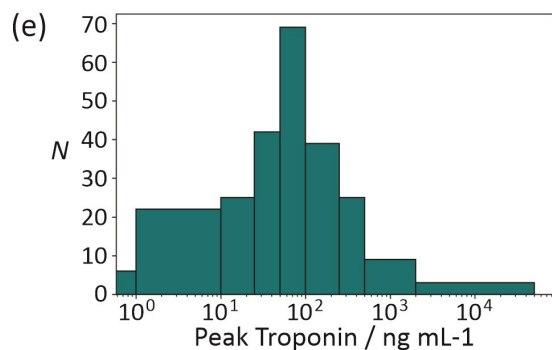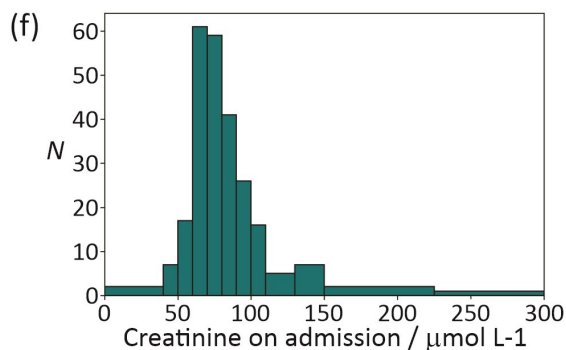

Supplement: AN-150-D5AN00565E-s001 [file AN-150-D5AN00565E-s001.pdf]

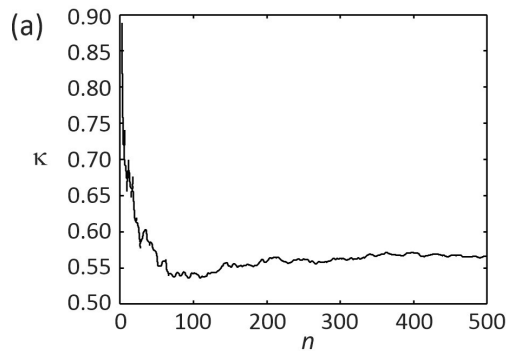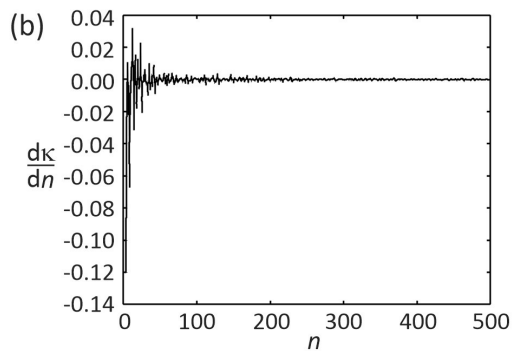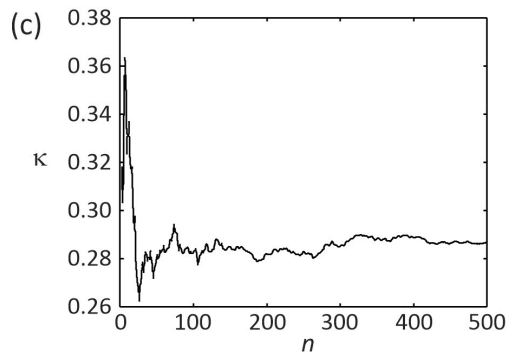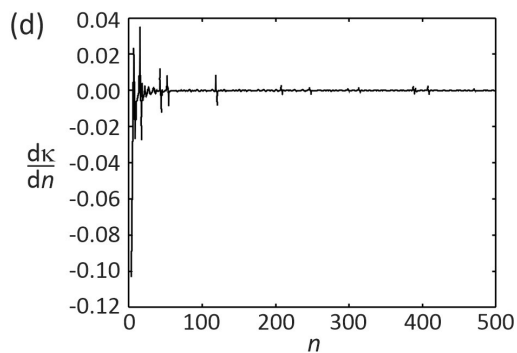

Supplement: AN-150-D5AN00565E-s002 [file AN-150-D5AN00565E-s002.pdf]

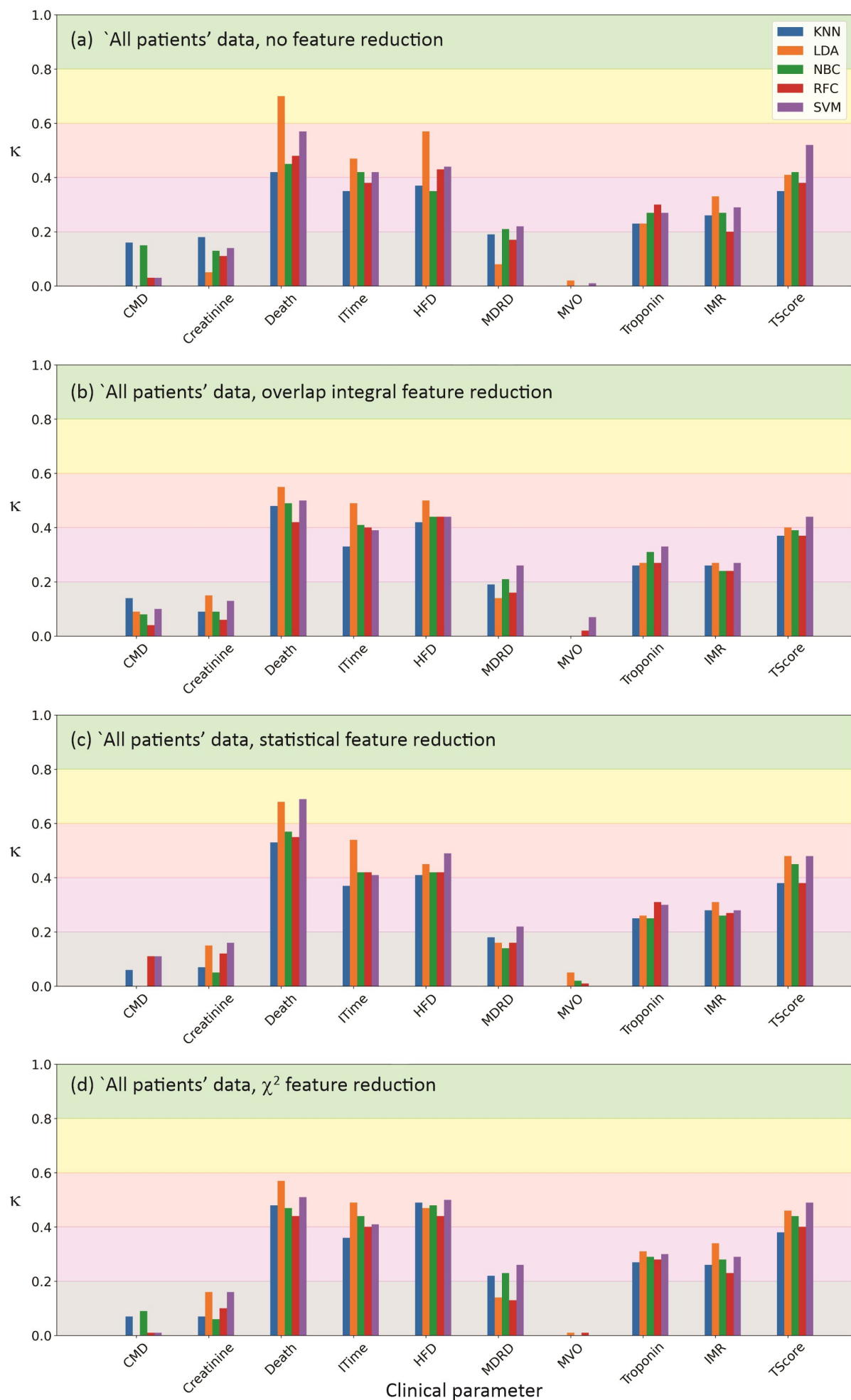

Supplement: AN-150-D5AN00565E-s003 [file AN-150-D5AN00565E-s003.pdf]

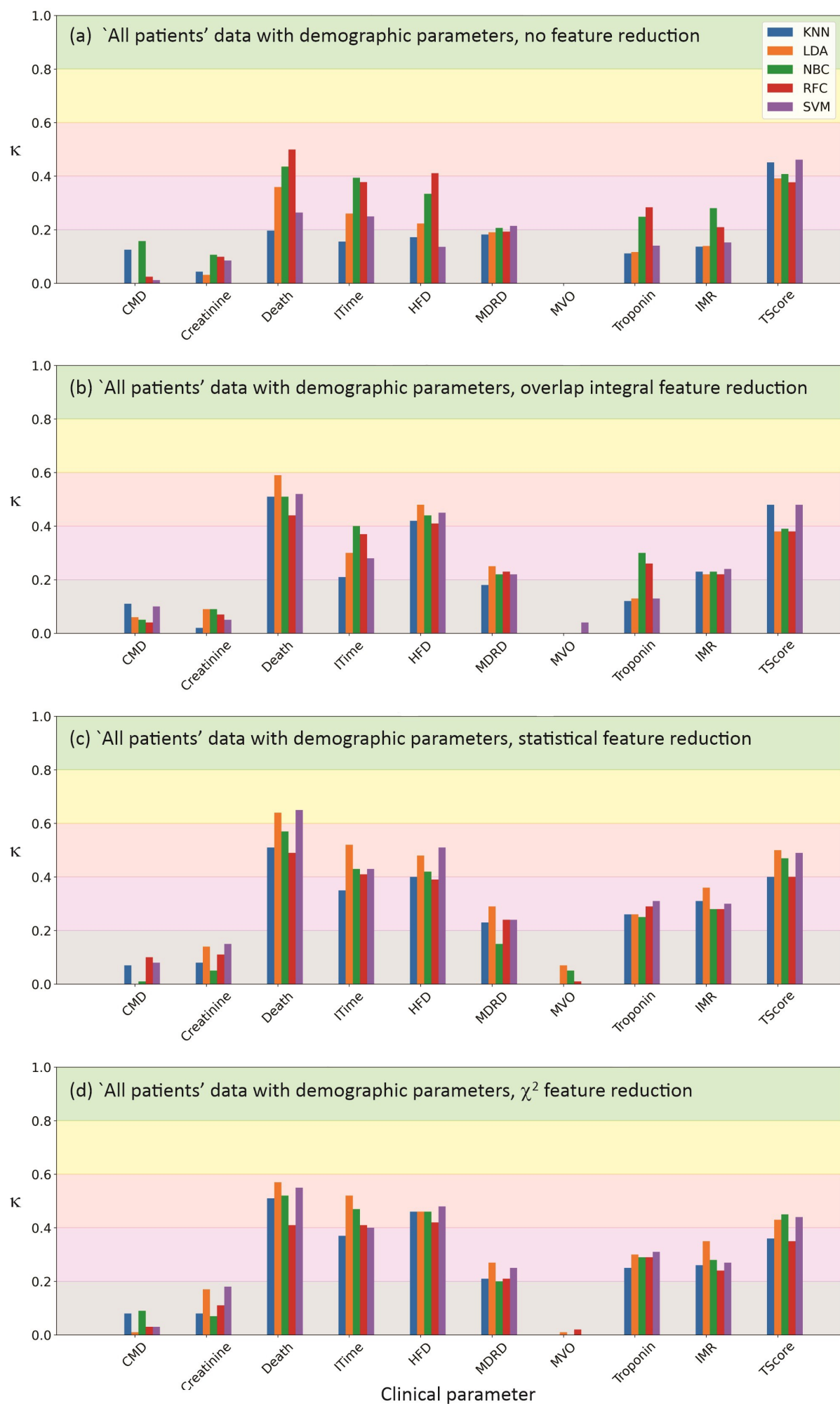

Supplement: AN-150-D5AN00565E-s004 [file AN-150-D5AN00565E-s004.pdf]

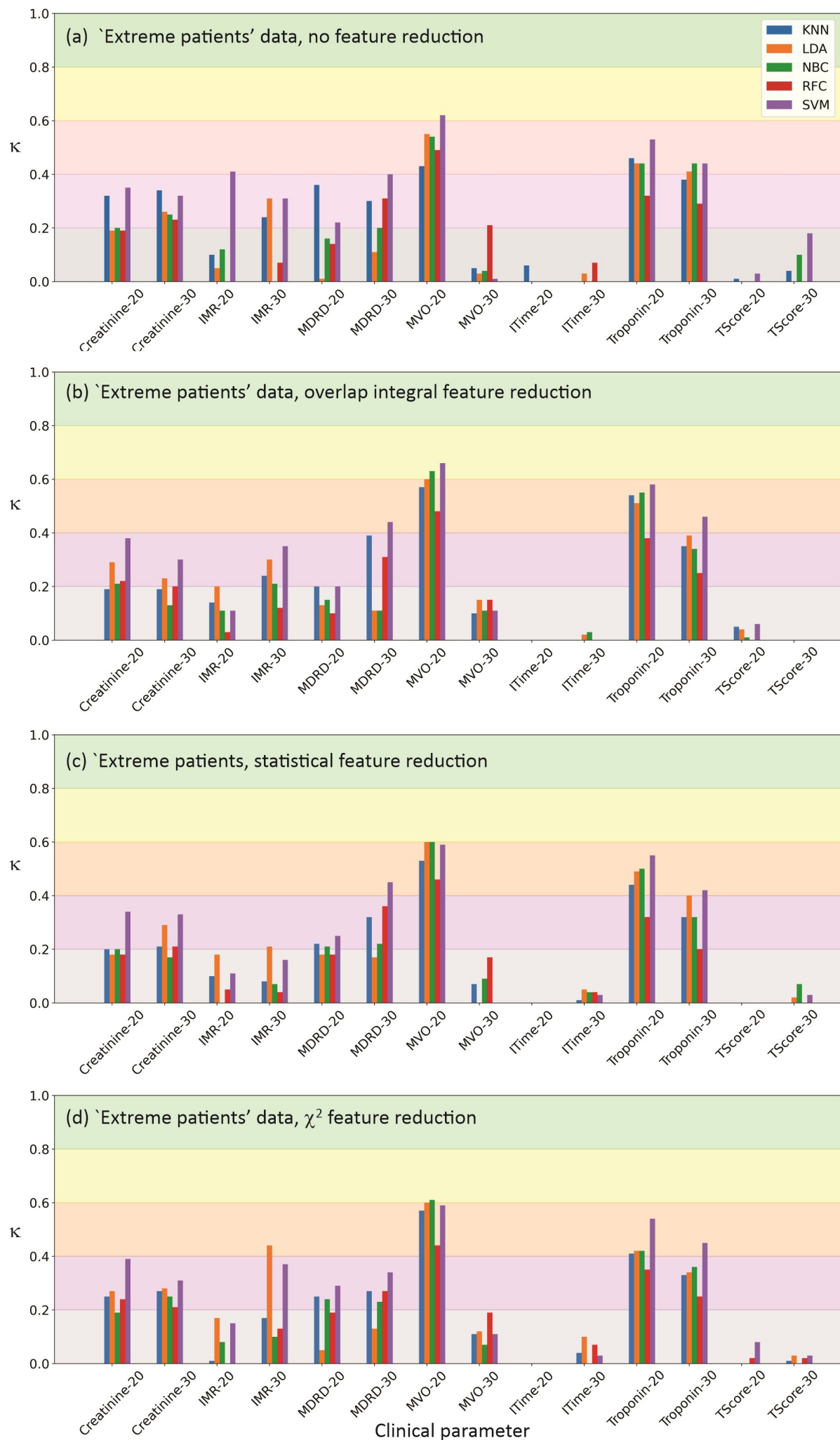

Supplement: AN-150-D5AN00565E-s005 [file AN-150-D5AN00565E-s005.pdf]

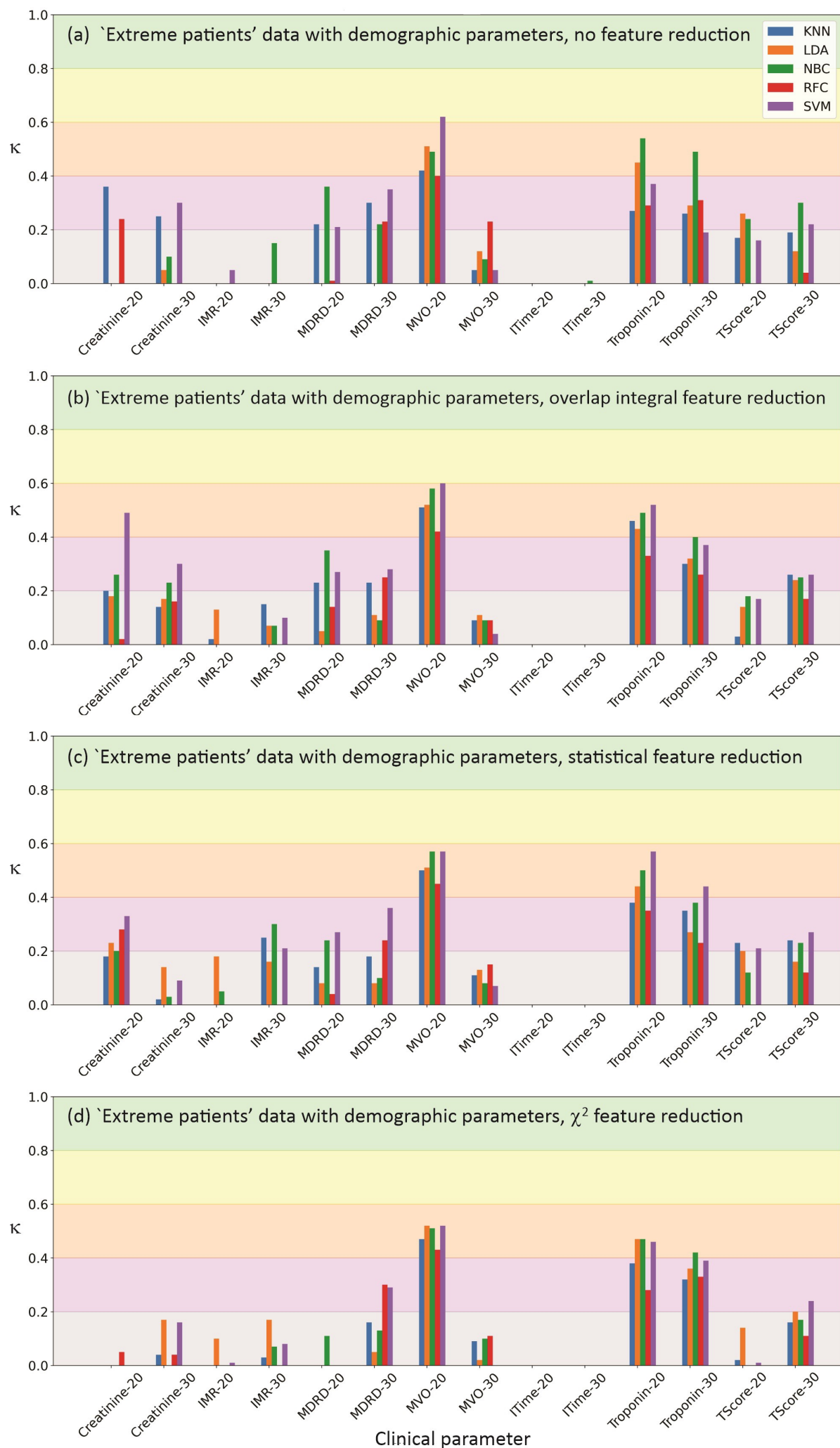

Supplement: AN-150-D5AN00565E-s006 [file AN-150-D5AN00565E-s006.pdf]
