# Supplementary material for: Prediction of clinical outcomes of ST-elevated myocardial infarction patients using atmospheric solids analysis probe mass spectrometry and machine learning
Source: Analyst. 2025 Sep 24;150(22):4982–96. doi: 10.1039/d5an00565e (PMC12498272; doi:10.1039/d5an00565e)
Supplement: AN-150-D5AN00565E-s007 [file AN-150-D5AN00565E-s007.pdf]

Supplementary Information (SI) for Analyst.
This journal is © The Royal Society of Chemistry 2025

Supporting Information: Prediction of clinical outcomes of ST-elevated myocardial infarction patients using atmospheric solids analysis probe mass spectrometry and machine learning

Annabel S. J. Eardley-Brunt[a], Thomas Mills[a], Rafail Kotronias[(b),(c)], Giovanni Luigi de Maria[(b),(c)], Keith Channon[(b),(c)], The Oxford Acute Myocardial Infarction (OxAMI) study, and Claire Vallance[a]

(a) Department of Chemistry, University of Oxford, Chemistry Research Laboratory, 12 Mansfield Rd, Oxford OX1 3TA, UK
(b) NIHR Oxford Biomedical Research Centre, John Radcliffe Hospital, Oxford University Hospitals, Oxford, UK
(c) Division of Cardiovascular Medicine, Radcliffe Department of Medicine, University of Oxford, Oxford, UK

1 Patient population distributions for each of the clinical parameters of interest

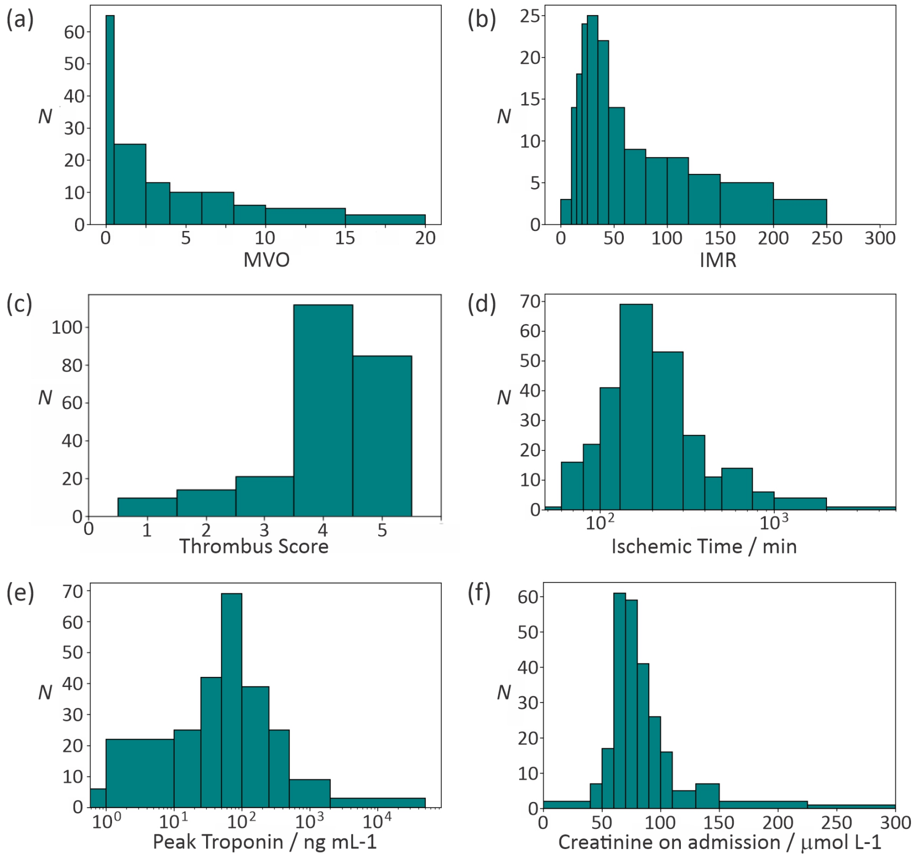

Figure 1: Histogram plots of the patient population distributions across each of the clinical parameters of interest: (a) MVO measured six months after the pPCI; (b) IMR measured during the pPCI; (c) Thrombus score; (d) ITime prior to the pPCI; (e) peak troponin level measured prior to pPCI; (f) creatinine level.

2 Thresholds for classification analysis

Most of the clinical parameters investigated are continuous, in the sense that they can take a broad range of numerical values. As explained in Section 2.4 of the main article, the individual values

for each variable have been grouped into classes (mostly by defining two groups corresponding to ‘high’ and ‘low’ values for each parameter) so that classification algorithms can be employed. The threshold values separating the groups for each variable have been chosen either by using definitions from the literature (ischemic time (ITime) and IMR), or using the median measured value where no threshold is defined (peak troponin and creatinine on admission). Thrombus score (TScore) was compressed from a five-class grouping (TScore 1, 2, 3, 4, and 5) to a binary grouping (TScore 1-3 and TScore 4-5) due to low populations in the low thrombus score classes. Ischemic time (ITime) was grouped by ITime <6 hours and ITime >6 hours as defined by J. A. Rodrigues *et al.*[*]. The index of microcirculatory resistance (IMR) is grouped by IMR<40 and IMR>40 as determined by W. Fearon *et al.* and G. L. De Maria *et al.*[†]

3 Machine learning classification methods

The five machine learning classification models were optimised within the MATLAB programming environment. Each classifier has a set of ‘hyperparameters’ that must be optimised alongside training the algorithm using the labelled training data set. In our case the training data used for each model comprised a set of plasma mass spectra from patients enrolled in the OxAMI study, with each mass spectrum being labelled with the value of one of the clinical parameters of interest for the corresponding patient. The relevant hyperparameters for each algorithm are listed in Table 1. For example, the KNN algorithm treats each mass spectrum as a vector in a multidimensional space, and assigns a label for an ‘unknown’ mass spectrum by identifying the labels of the nearest neighbour mass spectra used to train the algorithm. The hyperparameters in this case are the number of nearest neighbours to consider, and the distance metric used to identify the nearest neighbours.

The models were trained within MATLAB using the functions `fitcknn`, `fitcsvm`, `fitcdiscr`, `fitcnb`, and `fitcensemble`, for the KNN, SVM, LDA, NBC, and RFC algorithms, respectively. Hyperparameter optimisation was conducted for each model using the MATLAB argument `OptimiseHyperparameters` for each function with the `'auto'` keyword, prior to completing the classification analysis.

Table 1: Hyperparameter optimisation for machine learning classification models.

Model	Hyperparameters
K-nearest neighbours	Number of neighbours<br>Distance metric
Support vector machines	Box constraint<br>Kernel Scale
Linear discriminant analysis	Delta<br>Gamma
Naïve Bayes classifier	Distribution names<br>Width
Random forest classifier	Method<br>Number of learning cycles<br>Learn rate<br>For tree method: minimum leaf size<br>For discriminant method: Delta and gamma<br>For KNN method: Distance and Number of neighbours

[*]Rodrigues *et al.*, Independent predictors of late presentation in patients with ST-Segment elevation myocardial infarction, *Arquivos Varsileiros de Cardiologia*, **111**, 587-593 (2018).

[†]W. F. Fearon *et al.*, Prognostic value of the index of microcirculatory resistance measured after primary percutaneous coronary intervention, *Circulation*, **127**, 2436-2441 (2013); . G. L. de Maria *et al.*, How does coronary stent implantation impact on the status of the microcirculation during primary percutaneous coronary intervention in patients with ST-elevation myocardial infarction? *Eur. Heart J.*, **36**, 3165-3177 (2015).

4 Optimisation of the ML cross-validation partition number

The number of CV partitions to use was optimised by determining the point at which the κ score obtained over each classification analysis converged on an approximately constant value. A study was conducted in which each of the five machine learning models were used to assess each categorical clinical variable over 500 partitions. Each partition and model combination generates a confusion matrix, which were stacked by model. A κ score was determined for the confusion matrix resulting from the addition of each new partition. For each different machine learning model across each clinical parameter, the convergence in κ was found to be between 200 and 300 partitions. A pair of examples are shown in Figure 2 for a small sample set (patient mortality) and a large sample set (peak troponin). In the interest of computational time, 200 partitions were used.

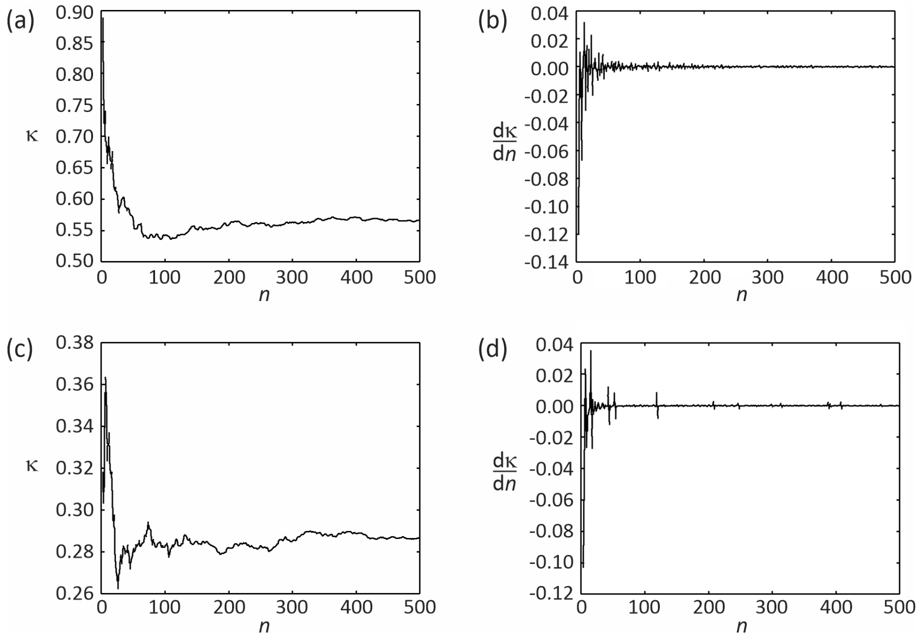

Figure 2: Dependence of κ score on the number of data partitions, n, included in the analysis: (a) κ and (b) $d\kappa/dn$ plotted as a function of n for patient mortality; (c) κ and (d) $d\kappa/dn$ plotted as a function of n for peak tropinin.

5 Machine learning results for classification of 'All patients' clinical data

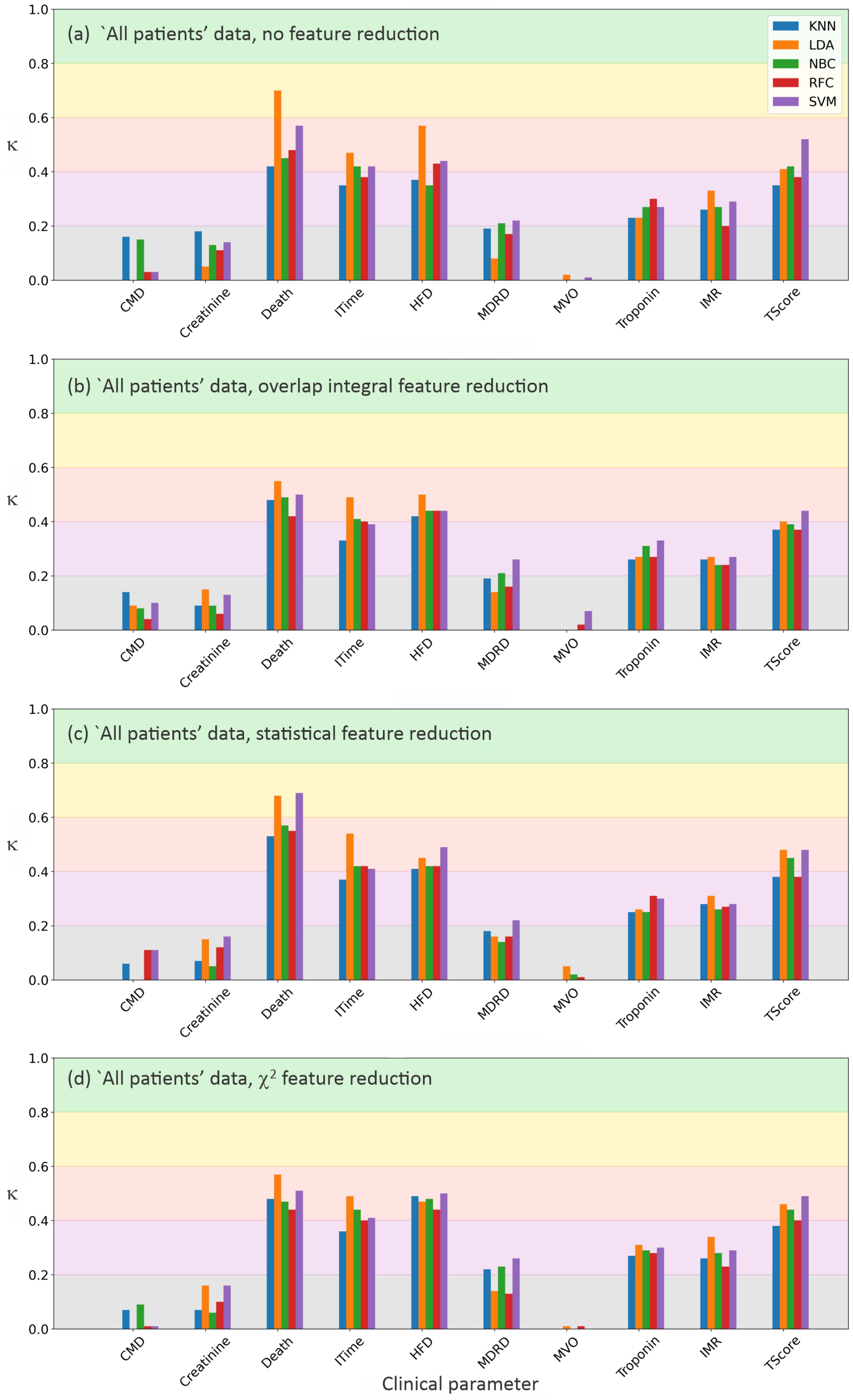

Figure 3: The κ scores obtained from analysing 'All patients' data set with: (a) no feature reduction; (b) feature reduction with overlap integral method; (c) feature reduction with statistical method; and (d) feature reduction by χ^2 method. The coloured vertical bars show the κ score obtained for each ML model for the clinical variable indicated. The horizontal coloured bars show the level of agreement, with *no agreement* in grey, *low agreement* in purple, *fair agreement* in light red, *moderate agreement* in yellow, and *strong agreement* in green.

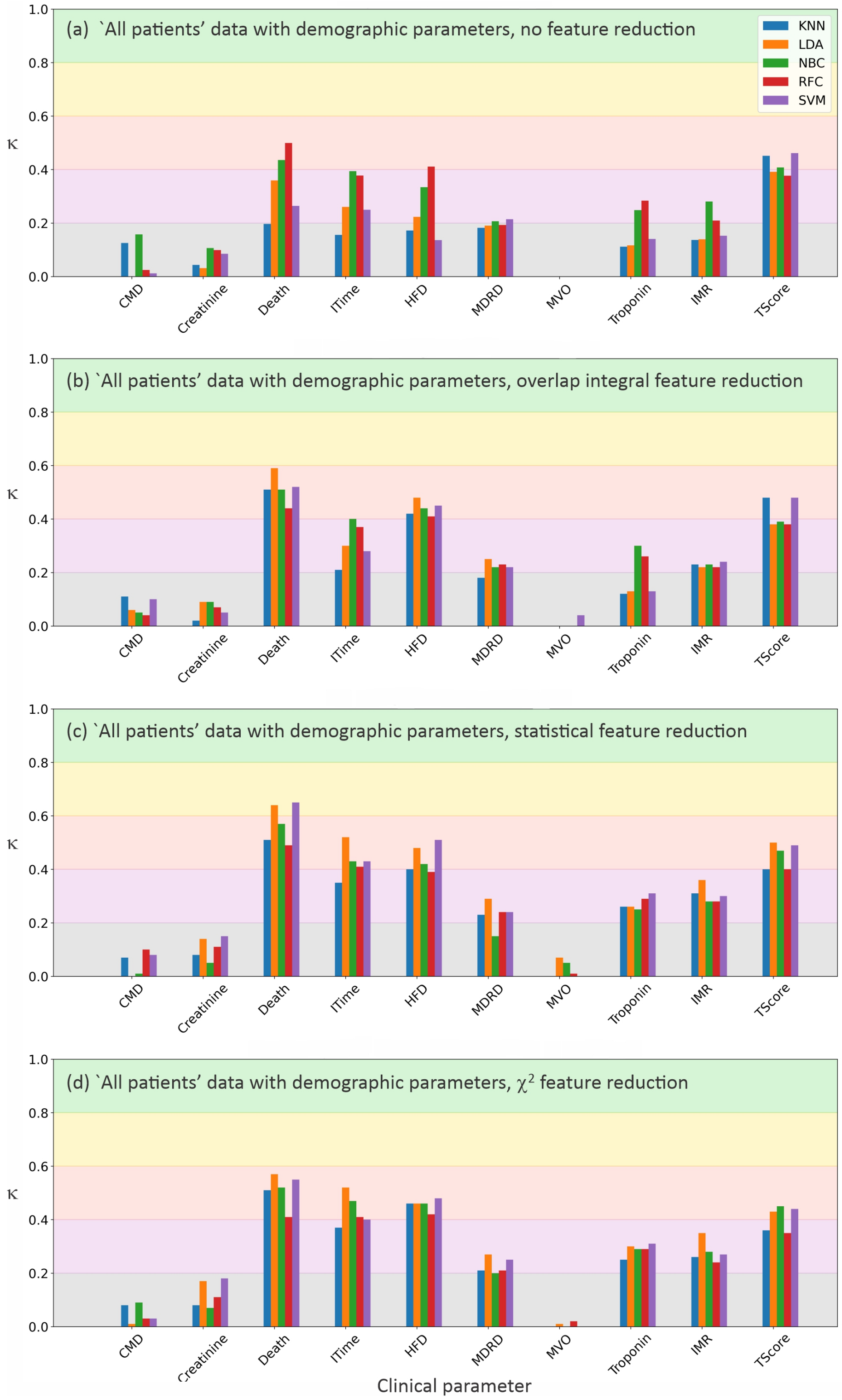

Figure 4: The κ scores obtained from analysing 'All patients' data set with the inclusion of demographic data, and: (a) no feature reduction; (b) feature reduction with overlap integral method; (c) feature reduction with statistical method; and (d) feature reduction by χ^2 method. The horizontal coloured bars show the level of agreement with *no agreement* in grey, *low agreement* in purple, *fair agreement* in light red, *moderate agreement* in yellow, and *strong agreement* in green.

6 Machine learning results for classification of ‘extreme patients’ clinical data

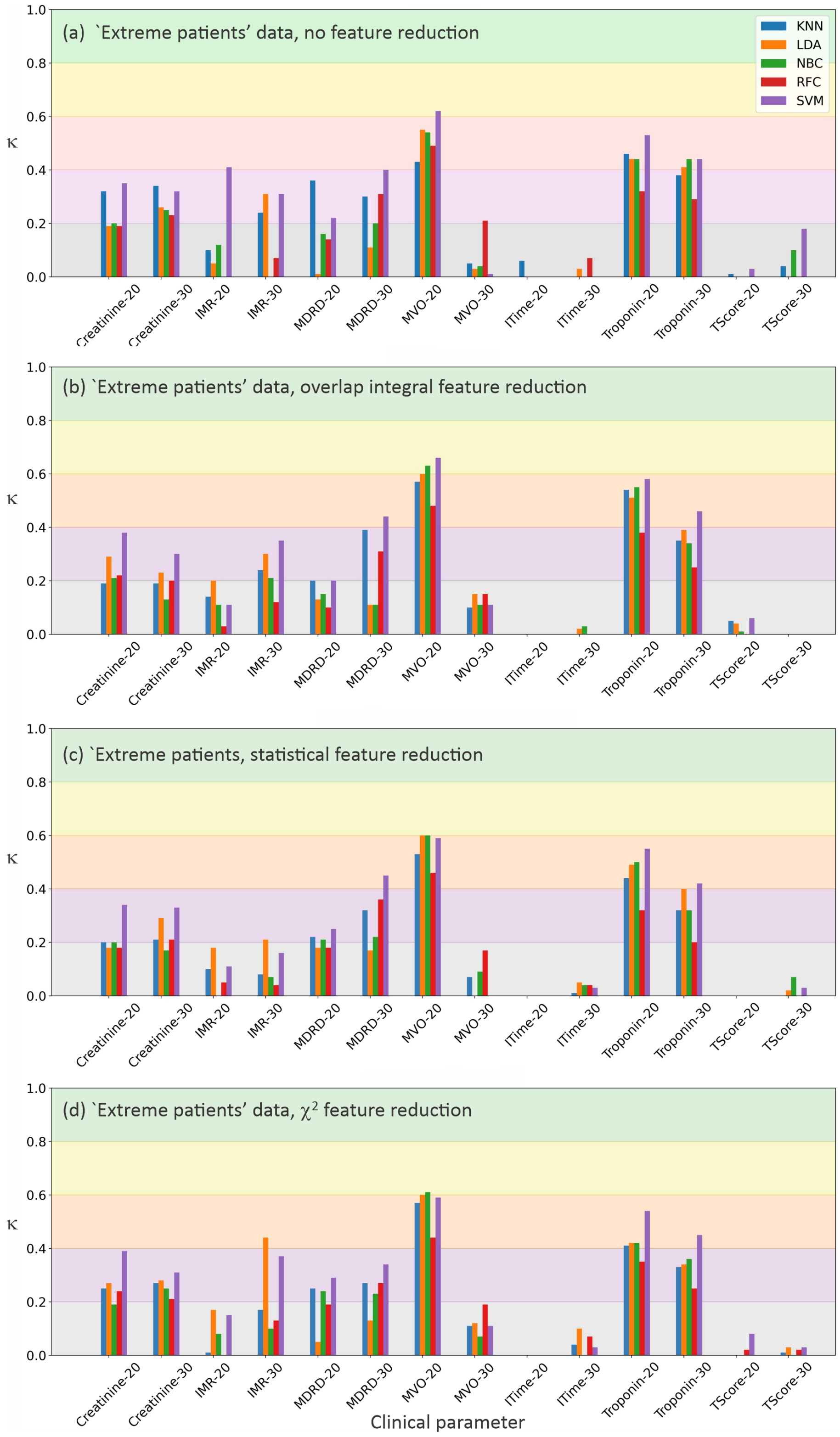

Figure 5: The κ scores obtained from analysing the ‘Extreme patients’ data sets, with (a) no feature reduction; (b) feature reduction with overlap integral method; (c) feature reduction with statistical method; and (d) feature reduction by χ^2 method. The coloured vertical bars show the κ score obtained for each ML model for that clinical variable. The horizontal coloured bars show the level of agreement with *no agreement* in grey, *low agreement* in purple, *fair agreement* in light red, *moderate agreement* in yellow, and *strong agreement* in green.

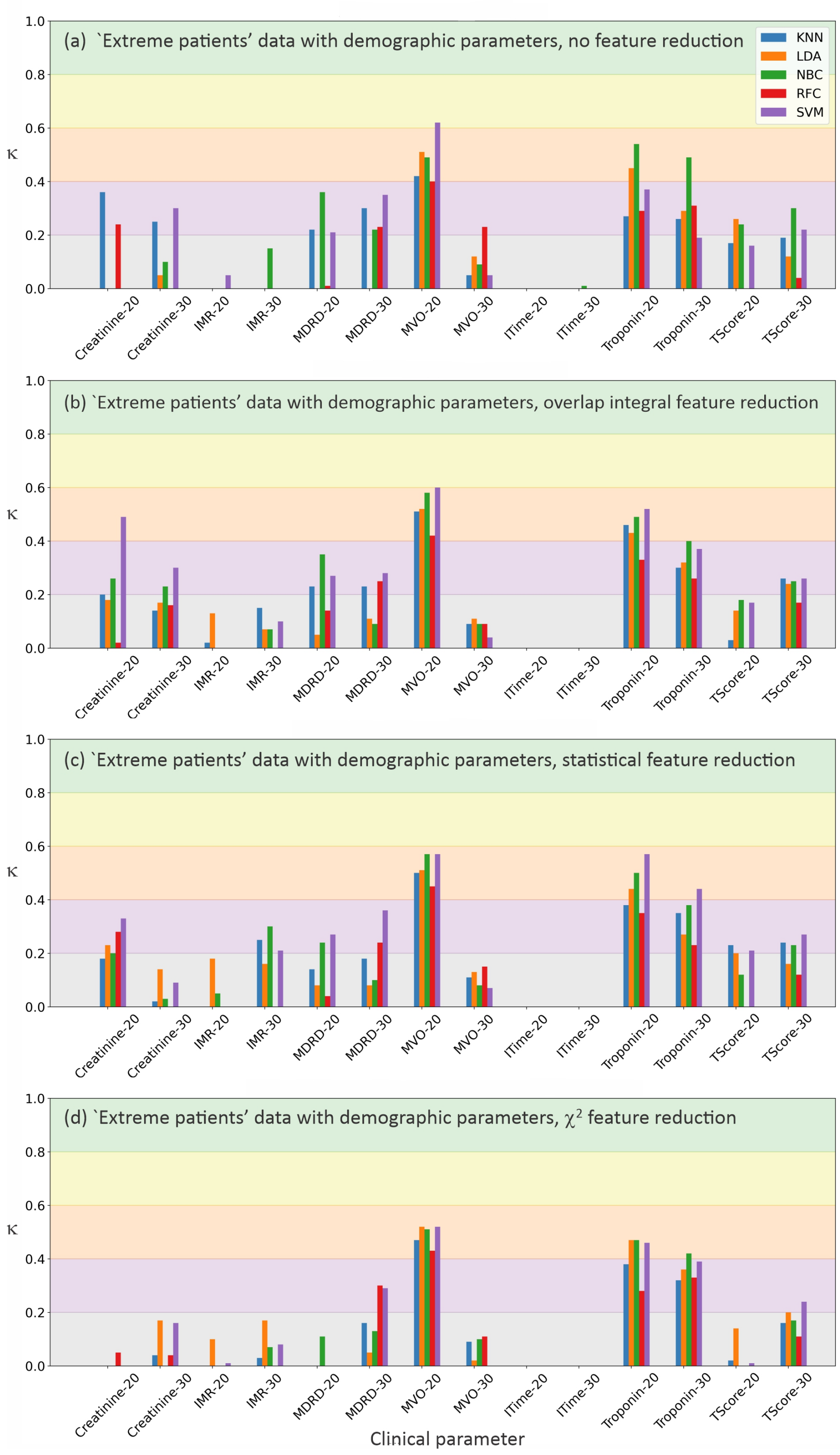

Figure 6: The κ scores obtained from analysing the 'Extreme patients' data sets with inclusion of clinical data and: (a) no feature reduction; (b) feature reduction with overlap integral method; (c) feature reduction with statistical method; and (d) feature reduction by χ^2 method. The coloured vertical bars show the κ score obtained for each ML model for that clinical variable. The horizontal coloured bars show the level of agreement with *no agreement* in grey, *low agreement* in purple, *fair agreement* in light red, *moderate agreement* in yellow, and *strong agreement* in green.

7 Best-performing machine learning models for classification of ‘All patients’ data

Table 2: The top κ scores obtained for each clinical variable investigated across the ‘All patients’ data sets using different feature reduction methods.

Clinical variable	Maximum κ	Feature reduction	Clinical inputs used	Model
Mortality	0.70	None	No	LDA
HF	0.57	None	No	LDA
MVO	0.07	Statistics	Yes	LDA
IMR	0.36	Statistics	Yes	LDA
ITime	0.54	Statistics	No	LDA
Troponin	0.33	OI	No	SVM
Creatinine	0.18	None	No	KNN

8 Best-performing machine learning models for classification of ‘Extreme patients’ data

Table 3: The top κ scores obtained for each clinical variable investigated across the ‘extreme patients’ data sets using different feature reduction methods.

Clinical variable	maximum kappa	Feature reduction	Clinical input	Model
MVO-20	0.66	Statistics	No	SVM
MVO-30	0.23	None	Yes	RFC
IMR-20	0.41	None	No	SVM
IMR-30	0.44	OI	No	LDA
TScore-20	0.26	None	Yes	LDA
TScore-30	0.30	None	Yes	NBC
ITime-20	0.06	None	No	KNN
ITime-30	0.10	OI	No	LDA
Troponin-20	0.58	Statistics	No	SVM
Troponin-30	0.49	None	Yes	NBC
Creatinine-20	0.49	Statistics	Yes	SVM
Creatinine-30	0.34	None	No	KNN

9 Full results of machine learning analysis for classification of ‘All patients’ data

Table 4: Machine learning results for the classification of ‘All patients’ data.

Clinical variable	KNN kappa	SVM kappa	LDA kappa	NBC kappa	RFC kappa
**Mortality**	0.448±0.249	0.591±0.229	0.706±0.212	0.437±0.268	0.493±0.274
**HF**	0.4±0.264	0.405±0.247	0.544±0.257	0.347±0.263	0.393±0.278
**MVO**	-0.041±0.183	0.004±0.168	0.008±0.169	-0.04±0.148	-0.001±0.184
**IMR**	0.242±0.173	0.281±0.179	0.324±0.17	0.257±0.172	0.225±0.168
**Tscore**	0.337±0.201	0.54±0.184	0.382±0.189	0.411±0.193	0.383±0.203
**ITime**	0.308±0.201	0.439±0.196	0.448±0.214	0.396±0.197	0.387±0.218
**Creatinine**	0.158±0.134	0.153±0.113	0.053±0.128	0.121±0.128	0.089±0.14
**Troponin**	0.218±0.142	0.262±0.127	0.223±0.139	0.252±0.131	0.265±0.14

Table 5: Machine learning results for the classification of ‘All patients’ data with clinical input variables.

Clinical variable	KNN kappa	SVM kappa	LDA kappa	NBC kappa	RFC kappa
**Mortality**	0.384±0.273	0.565±0.269	-0.111±0	0.377±0.267	0.43±0.289
**HF**	0.41±0.281	0.397±0.272	0±0	0.291±0.243	0.417±0.266
**MVO**	-0.026±0.18	0.031±0.165	0±0	-0.02±0.16	0.002±0.198
**IMR**	0.23±0.176	0.305±0.17	-0.04±0	0.273±0.187	0.201±0.197
**Tscore**	0.358±0.194	0.498±0.175	0±0	0.444±0.201	0.381±0.194
**ITime**	0.324±0.186	0.433±0.192	0±0	0.481±0.223	0.409±0.208
**Troponin**	0.221±0.142	0.279±0.134	0±0	0.248±0.115	0.275±0.125
**Creatinine**	0.186±0.136	0.172±0.135	0±0	0.123±0.152	0.102±0.132

Table 6: Machine learning results for the classification of ‘All patients’ data with FR feature selection.

Clinical variable	KNN kappa	SVM kappa	LDA kappa	NBC kappa	RFC kappa
**Mortality**	0.456±0.269	0.496±0.286	0.552±0.265	0.487±0.274	0.415±0.278
**HF**	0.485±0.271	0.505±0.243	0.472±0.258	0.484±0.239	0.458±0.262
**MVO**	-0.004±0.189	-0.042±0.194	0.007±0.177	-0.018±0.172	-0.014±0.189
**IMR**	0.232±0.173	0.238±0.179	0.31±0.168	0.255±0.164	0.223±0.18
**Tscore**	0.388±0.202	0.49±0.192	0.458±0.193	0.456±0.195	0.379±0.204
**ITime**	0.38±0.19	0.413±0.202	0.506±0.179	0.443±0.183	0.395±0.216
**Troponin**	0.262±0.146	0.297±0.13	0.304±0.129	0.303±0.134	0.28±0.135
**Creatinine**	0.065±0.139	0.164±0.135	0.141±0.124	0.045±0.143	0.118±0.128

Table 7: Machine learning results for the classification of 'All patients' data with FR feature selection and clinical input variables.

Clinical variable	KNN kappa	SVM kappa	LDA kappa	NBC kappa	RFC kappa
**Mortality**	0.49±0.278	0.558±0.251	0.59±0.262	0.52±0.242	0.457±0.283
**HF**	0.456±0.253	0.458±0.24	0.487±0.25	0.45±0.224	0.428±0.251
**MVO**	-0.002±0.191	-0.01±0.199	0.031±0.197	0.013±0.189	0.03±0.202
**IMR**	0.248±0.191	0.246±0.186	0.334±0.171	0.261±0.171	0.255±0.188
**Tscore**	0.397±0.2	0.463±0.212	0.465±0.177	0.446±0.19	0.371±0.211
**ITime**	0.364±0.173	0.411±0.185	0.503±0.171	0.451±0.182	0.413±0.208
**Troponin**	0.248±0.154	0.288±0.143	0.29±0.138	0.273±0.145	0.288±0.14
**Creatinine**	0.083±0.129	0.182±0.137	0.176±0.135	0.053±0.132	0.109±0.127

Table 8: Machine learning results for the classification of 'All patients' data with OI feature selection.

Clinical variable	KNN kappa	SVM kappa	LDA kappa	NBC kappa	RFC kappa
**Mortality**	0.444±0.282	0.477±0.28	0.536±0.296	0.454±0.284	0.38±0.293
**HF**	0.391±0.255	0.402±0.245	0.454±0.267	0.431±0.261	0.389±0.271
**MVO**	-0.026±0.195	0.035±0.174	-0.023±0.187	-0.023±0.192	-0.008±0.199
**IMR**	0.246±0.181	0.262±0.17	0.27±0.165	0.216±0.183	0.241±0.179
**Tscore**	0.387±0.212	0.446±0.202	0.397±0.186	0.385±0.194	0.348±0.217
**ITime**	0.332±0.188	0.417±0.199	0.487±0.196	0.416±0.191	0.397±0.202
**Troponin**	0.268±0.143	0.309±0.14	0.278±0.138	0.316±0.147	0.29±0.128
**Creatinine**	0.095±0.139	0.143±0.123	0.154±0.14	0.076±0.143	0.062±0.129

Table 9: Machine learning results for the classification of 'All patients' data with OI feature selection and clinical input variables.

Clinical variable	KNN kappa	SVM kappa	LDA kappa	NBC kappa	RFC kappa
**Mortality**	0.47±0.3	0.49±0.285	0.576±0.289	0.499±0.266	0.439±0.276
**HF**	0.431±0.243	0.453±0.226	0.506±0.265	0.457±0.24	0.432±0.259
**MVO**	-0.013±0.199	0.027±0.162	-0.03±0.184	-0.034±0.177	0.004±0.191
**IMR**	0.2±0.151	0.229±0.155	0.216±0.168	0.243±0.173	0.231±0.18
**Tscore**	0.463±0.193	0.464±0.184	0.378±0.186	0.385±0.203	0.364±0.202
**ITime**	0.22±0.184	0.287±0.166	0.321±0.186	0.409±0.199	0.392±0.206
**Troponin**	0.124±0.124	0.141±0.135	0.135±0.129	0.303±0.122	0.28±0.143
**Creatinine**	0.034±0.127	0.064±0.14	0.108±0.125	0.088±0.142	0.068±0.127

Table 10: Machine learning results for the classification of 'All patients' data with statistical feature selection.

Clinical variable	KNN kappa	SVM kappa	LDA kappa	NBC kappa	RFC kappa
**Mortality**	0.488±0.281	0.676±0.236	0.656±0.224	0.542±0.245	0.523±0.254
**HF**	0.392±0.267	0.484±0.251	0.51±0.257	0.439±0.257	0.469±0.268
**MVO**	-0.001±0.195	-0.005±0.183	0.073±0.178	0.045±0.194	0.026±0.178
**IMR**	0.333±0.188	0.308±0.164	0.322±0.178	0.278±0.178	0.268±0.182
**Tscore**	0.387±0.188	0.452±0.181	0.467±0.2	0.447±0.208	0.397±0.208
**ITime**	0.334±0.205	0.398±0.182	0.536±0.189	0.428±0.207	0.396±0.201
**Troponin**	0.256±0.137	0.319±0.128	0.251±0.131	0.266±0.112	0.305±0.129
**Creatinine**	0.069±0.116	0.154±0.132	0.134±0.117	0.049±0.125	0.111±0.133

Table 11: Machine learning results for the classification of 'All patients' data with statistical feature selection and clinical input variables.

Clinical variable	KNN kappa	SVM kappa	LDA kappa	NBC kappa	RFC kappa
**Mortality**	0.509±0.298	0.685±0.239	0.645±0.25	0.591±0.247	0.558±0.269
**HF**	0.392±0.248	0.489±0.267	0.493±0.237	0.406±0.242	0.397±0.273
**MVO**	-0.013±0.194	-0.025±0.208	0.079±0.167	0.046±0.19	0.026±0.192
**IMR**	0.29±0.195	0.272±0.179	0.346±0.155	0.269±0.175	0.275±0.171
**Tscore**	0.39±0.202	0.474±0.172	0.497±0.18	0.468±0.203	0.403±0.216
**ITime**	0.349±0.2	0.399±0.193	0.531±0.172	0.424±0.197	0.406±0.198
**Troponin**	0.246±0.14	0.305±0.127	0.242±0.124	0.244±0.127	0.294±0.13
**Creatinine**	0.068±0.125	0.158±0.143	0.136±0.123	0.043±0.13	0.108±0.131

10 Full machine learning results for classification of 'Extreme patients' data

Table 12: Machine learning results for the classification of 'Extreme patients' data.

Clinical variable	KNN kappa	SVM kappa	LDA kappa	NBC kappa	RFC kappa
MVO-20	0.436±0.279	0.646±0.259	0.589±0.274	0.533±0.285	0.464±0.306
MVO-30	0.057±0.248	0.015±0.232	0.054±0.217	0.05±0.248	0.239±0.283
IMR-20	0.125±0.342	0.388±0.314	0.063±0.322	0.1±0.291	0.014±0.354
IMR-30	0.263±0.336	0.305±0.291	0.333±0.296	-0.005±0.312	0.04±0.349
TScore-20	0.03±0.315	0.01±0.295	-0.063±0.239	-0.02±0.226	-0.065±0.319
TScore-30	-0.001±0.262	0.14±0.242	-0.011±0.209	0.079±0.222	-0.054±0.256
ITime-20	0.081±0.335	-0.111±0.31	-0.03±0.273	-0.064±0.278	-0.07±0.314
ITime-30	-0.007±0.272	-0.115±0.193	-0.03±0.22	-0.065±0.238	0.034±0.27
Troponin-20	0.47±0.317	0.51±0.28	0.438±0.291	0.454±0.275	0.318±0.309
Troponin-30	0.379±0.272	0.46±0.23	0.413±0.251	0.445±0.245	0.253±0.254
Creatinine-20	0.334±0.302	0.373±0.294	0.26±0.326	0.23±0.31	0.217±0.329
Creatinine-30	0.339±0.254	0.35±0.239	0.271±0.262	0.213±0.262	0.183±0.294

Table 13: Machine learning results for the classification of 'Extreme patients' data with clinical input variables.

Clinical variable	KNN kappa	SVM kappa	LDA kappa	NBC kappa	RFC kappa
MVO-20	0.393±0.324	0.602±0.294	0.546±0.279	0.501±0.305	0.418±0.303
MVO-30	0.078±0.233	0.088±0.256	0.119±0.262	0.065±0.255	0.194±0.306
IMR-20	-0.016±0.318	0.029±0.324	-0.045±0.244	0.017±0.234	-0.19±0.332
IMR-30	0±0.301	-0.1±0.317	-0.035±0.307	0.085±0.344	-0.205±0.44
TScore-20	0.153±0.52	0.187±0.506	0.201±0.499	0.229±0.489	-0.13±0.484
TScore-30	0.282±0.378	0.233±0.332	0.052±0.305	0.355±0.358	0.088±0.351
ITime-20	-0.063±0.38	-0.254±0.219	-0.195±0.282	-0.238±0.281	-0.15±0.364
ITime-30	-0.127±0.307	-0.088±0.193	-0.108±0.247	0.006±0.277	0.022±0.369
Creatinine-20	0.37±0.629	-0.055±0.391	-0.035±0.272	-0.07±0.407	0.16±0.661
Creatinine-30	0.272±0.365	0.302±0.342	0.067±0.249	0.091±0.304	0.021±0.339
Troponin-20	0.312±0.386	0.354±0.382	0.41±0.371	0.522±0.355	0.222±0.389
Troponin-30	0.268±0.305	0.227±0.298	0.254±0.299	0.491±0.307	0.339±0.322

Table 14: Machine learning results for the classification of 'Extreme patients' data with FR feature selection.

Clinical variable	KNN kappa	SVM kappa	LDA kappa	NBC kappa	RFC kappa
MVO-20	0.502±0.298	0.554±0.302	0.562±0.27	0.574±0.278	0.478±0.31
MVO-30	0.058±0.261	-0.006±0.259	0.062±0.26	0.109±0.289	0.125±0.273
IMR-20	0.059±0.336	0.14±0.315	0.16±0.324	-0.003±0.298	0.071±0.332
IMR-30	0.066±0.315	0.156±0.364	0.198±0.323	0.124±0.335	0.023±0.324
TScore-20	-0.074±0.323	-0.063±0.34	-0.056±0.292	-0.015±0.338	-0.075±0.331
TScore-30	-0.022±0.273	0.018±0.29	0.007±0.279	0.078±0.286	-0.073±0.262
ITime-20	-0.045±0.305	-0.06±0.331	-0.078±0.327	-0.063±0.336	-0.141±0.304
ITime-30	0.026±0.26	0.051±0.286	0.047±0.281	0.049±0.267	0.104±0.283
Creatinine-20	0.175±0.329	0.293±0.317	0.158±0.336	0.211±0.328	0.218±0.318
Creatinine-30	0.237±0.242	0.327±0.277	0.292±0.269	0.176±0.27	0.27±0.3
Troponin-20	0.448±0.299	0.57±0.271	0.503±0.272	0.493±0.289	0.36±0.306
Troponin-30	0.323±0.25	0.394±0.224	0.39±0.231	0.277±0.275	0.24±0.231

Table 15: Machine learning results for the classification of 'Extreme patients' data with FR feature selection and clinical input variables.

Clinical variable	KNN kappa	SVM kappa	LDA kappa	NBC kappa	RFC kappa
MVO-20	0.458±0.279	0.524±0.29	0.423±0.286	0.484±0.287	0.378±0.327
MVO-30	0.06±0.265	0.009±0.279	0.009±0.277	0.094±0.315	0.131±0.267
IMR-20	-0.057±0.365	0.003±0.369	0.139±0.426	-0.039±0.373	-0.178±0.376
IMR-30	0.14±0.549	0.155±0.471	0.115±0.503	0.22±0.56	-0.105±0.474
TScore-20	0.016±0.484	0.036±0.556	0.071±0.562	-0.026±0.561	-0.158±0.469
TScore-30	0.142±0.38	0.192±0.354	0.147±0.351	0.14±0.376	0.088±0.338
ITime-20	-0.162±0.362	-0.176±0.289	-0.078±0.418	-0.168±0.383	-0.174±0.389
ITime-30	-0.144±0.323	-0.124±0.291	-0.102±0.335	-0.139±0.328	-0.088±0.35
Troponin-20	0.408±0.366	0.498±0.366	0.429±0.37	0.436±0.352	0.334±0.375
Troponin-30	0.308±0.351	0.365±0.362	0.33±0.344	0.395±0.32	0.344±0.364
Creatinine-20	-0.235±0.584	-0.055±0.378	-0.13±0.494	0.005±0.526	0.06±0.685
Creatinine-30	0.034±0.362	0.162±0.363	0.1±0.39	0.04±0.359	0.079±0.378

Table 16: Machine learning results for the classification of 'Extreme patients' data with OI feature selection.

**Clinical variable**	**KNN kappa**	**SVM kappa**	**LDA kappa**	**NBC kappa**	**RFC kappa**
MVO-20	0.571±0.283	0.61±0.276	0.631±0.265	0.654±0.274	0.493±0.293
MVO-30	0.088±0.297	0.146±0.257	0.122±0.257	0.069±0.269	0.194±0.274
IMR-20	0.038±0.316	0.173±0.35	0.18±0.322	0.103±0.311	-0.056±0.311
IMR-30	0.199±0.306	0.335±0.349	0.404±0.337	0.06±0.347	0.143±0.375
TScore-20	-0.026±0.339	0.011±0.353	-0.088±0.325	-0.125±0.303	-0.056±0.316
TScore-30	0.032±0.278	0.05±0.253	0.028±0.241	-0.029±0.233	-0.003±0.256
ITime-20	-0.048±0.307	-0.166±0.318	-0.105±0.311	-0.14±0.294	-0.089±0.345
ITime-30	0.002±0.28	0.042±0.28	0.09±0.278	-0.005±0.285	0.071±0.274
Troponin-20	0.438±0.313	0.579±0.279	0.408±0.302	0.439±0.302	0.361±0.301
Troponin-30	0.341±0.263	0.443±0.234	0.324±0.265	0.379±0.253	0.226±0.248
Creatinine-20	0.253±0.37	0.412±0.326	0.324±0.376	0.139±0.332	0.273±0.372
Creatinine-30	0.27±0.276	0.33±0.255	0.293±0.273	0.232±0.256	0.185±0.282

Table 17: Machine learning results for the classification of 'Extreme patients' data with OI feature selection and clinical input variables.

**Clinical variable**	**KNN kappa**	**SVM kappa**	**LDA kappa**	**NBC kappa**	**RFC kappa**
MVO-20	0.503±0.301	0.562±0.301	0.536±0.271	0.537±0.283	0.414±0.292
MVO-30	0.129±0.27	0.073±0.315	0.115±0.318	0.076±0.308	0.175±0.313
IMR-20	-0.079±0.345	-0.076±0.361	0.133±0.442	0.023±0.383	-0.178±0.373
IMR-30	0.215±0.557	0.095±0.487	0.195±0.591	0.26±0.494	-0.14±0.437
TScore-20	0.172±0.534	0.246±0.453	0.176±0.581	0.139±0.486	-0.085±0.493
TScore-30	0.34±0.368	0.328±0.338	0.237±0.374	0.25±0.363	0.1±0.38
ITime-20	-0.18±0.378	-0.231±0.299	-0.239±0.361	-0.222±0.343	-0.216±0.401
ITime-30	-0.101±0.35	-0.071±0.268	-0.084±0.333	-0.212±0.296	-0.075±0.348
Troponin-20	0.48±0.398	0.57±0.397	0.492±0.363	0.522±0.35	0.388±0.396
Troponin-30	0.338±0.35	0.45±0.339	0.288±0.332	0.371±0.332	0.228±0.323
Creatinine-20	0.08±0.637	0.295±0.547	0.135±0.631	0.065±0.666	0.175±0.638
Creatinine-30	0.043±0.353	0.095±0.337	0.101±0.341	0.003±0.401	-0.005±0.313

Table 18: Machine learning results for the classification of 'Extreme patients' data with stats feature selection.

**Clinical variable**	**KNN kappa**	**SVM kappa**	**LDA kappa**	**NBC kappa**	**RFC kappa**
MVO-20	0.562±0.27	0.663±0.27	0.576±0.276	0.64±0.269	0.48±0.297
MVO-30	0.14±0.262	0.123±0.265	0.133±0.261	0.069±0.249	0.152±0.27
IMR-20	0.111±0.331	0.078±0.356	0.191±0.329	0.08±0.372	0.021±0.341
IMR-30	0.228±0.351	0.324±0.331	0.278±0.312	0.175±0.338	0.189±0.332
TScore-20	-0.006±0.318	0.03±0.353	0.008±0.305	-0.013±0.313	-0.023±0.325
TScore-30	-0.055±0.229	-0.064±0.213	-0.104±0.225	-0.067±0.23	-0.1±0.283
ITime-20	-0.038±0.298	-0.078±0.299	-0.076±0.298	-0.035±0.291	-0.079±0.322
ITime-30	-0.027±0.276	0.035±0.266	0.022±0.28	0.01±0.27	0.009±0.267
Troponin-20	0.521±0.311	0.561±0.284	0.483±0.28	0.55±0.282	0.351±0.288
Troponin-30	0.376±0.27	0.457±0.242	0.372±0.257	0.349±0.275	0.248±0.268
Creatinine-20	0.246±0.329	0.383±0.323	0.308±0.35	0.194±0.343	0.183±0.329
dCreatinine-30	0.234±0.262	0.333±0.241	0.243±0.259	0.145±0.245	0.17±0.262

Table 19: Machine learning results for the classification of 'Extreme patients' data with stats feature selection with clinical input variables.

**Clinical variable**	**KNN kappa**	**SVM kappa**	**LDA kappa**	**NBC kappa**	**RFC kappa**
MVO-20	0.503±0.311	0.585±0.296	0.5±0.278	0.592±0.258	0.44±0.301
MVO-30	0.132±0.269	0.082±0.276	0.117±0.274	0.105±0.279	0.112±0.282
IMR-20	0.019±0.391	-0.018±0.431	0.101±0.44	0.002±0.441	0.054±0.467
IMR-30	0.165±0.509	0.085±0.547	0.08±0.637	0.09±0.513	-0.22±0.472
TScore-20	0.02±0.442	0.219±0.467	0.143±0.465	0.106±0.501	-0.106±0.453
TScore-30	0.245±0.345	0.288±0.353	0.215±0.364	0.208±0.373	0.163±0.398
ITime-20	-0.175±0.371	-0.136±0.329	-0.214±0.36	-0.139±0.358	-0.18±0.378
ITime-30	-0.08±0.322	-0.05±0.329	-0.064±0.369	-0.034±0.324	-0.059±0.335
Troponin-20	0.476±0.41	0.54±0.385	0.492±0.367	0.54±0.351	0.36±0.387
Troponin-30	0.335±0.339	0.399±0.348	0.353±0.299	0.37±0.318	0.311±0.349
Creatinine-20	0.255±0.73	0.44±0.623	0.2±0.73	0.31±0.69	-0.015±0.526
Creatinine-30	0.111±0.377	0.219±0.332	0.146±0.344	0.214±0.339	0.136±0.377

11 Significant m/z peaks determined through feature reduction

Table 20: The m/z ion peaks identified as being important for classification of patients according to each clinical parameter by all of the three feature reduction methods employed, with t statistic and p score. These data were generated from the 'All patients' data set, with the exception of the data for MVO, which was generated from the MVO-20 data set. See Figure 1 of the main article for box and whisker plots of the m/z peak intensity distributions for each variable.

**Clinical variable**	m/z **peaks**	t **statistic**	p **score**
Mortality	129	4.27	1.02×10^{-4}
	169	4.82	1.75×10^{-5}
	156	5.3	3.62×10^{-6}
	197	4.74	2.26×10^{-5}
HFD	208	4.73	2.00×10^{-5}
	219	2.79	7.59×10^{-3}
	532	5.45	1.73×10^{-6}
	189	-3.9	2.99×10^{-4}
MVO	355	4.77	2.71×10^{-5}
	387	-5.08	1.03×10^{-5}
	531	-4.06	2.37×10^{-4}
	532	-4.41	8.34×10^{-5}
	533	-3.97	3.04×10^{-4}
IMR	45	5.47	2.32×10^{-7}
	46	5.6	1.30×10^{-7}
	538	-2.94	3.89×10^{-3}
ITime	45	5.11	1.72×10^{-6}
	46	-5.72	1.29×10^{-7}
	387	4.65	1.11×10^{-4}
TScore	45	-5.64	1.89×10^{-7}
	46	-5.87	6.92×10^{-8}
	115	2.50	1.42×10^{-2}
Troponin	45	-2.65	8.63×10^{-3}
	46	4.38	1.82×10^{-5}
	49	3.38	8.71×10^{-4}
	370	-5.92	1.23×10^{-8}
	371	-5.72	3.50×10^{-8}
	576	4.84	2.50×10^{-6}
Creatinine	114	-2.91	3.91×10^{-3}
	62	-4.48	1.14×10^{-5}
